# The mycobacterial nucleoid-associated protein NapM exhibits stress-induced septal localization and modulates cell envelope gene expression

**DOI:** 10.1128/spectrum.03938-25

**Published:** 2026-06-09

**Authors:** Kornel Milcarz, Joanna Hołówka, Jakub Pawełczyk, Mariola Paściak, Yaroslav Lavrynchuk, Michał Tracz, Przemysław Płociński, Jolanta Zakrzewska-Czerwińska

**Affiliations:** 1 Department of Molecular Microbiology, University of Wrocław 49572, Wrocław, Poland; 2 Laboratory of Genetics and Physiology of Mycobacterium, Institute of Medical Biology, Polish Academy of Sciences 111423, Łódź, Poland; 3 Department of Immunology of Infectious Diseases, Hirszfeld Institute of Immunology and Experimental Therapy, Polish Academy of Sciences 89217, Wrocław, Poland; 4 Bio-Med-Chem Doctoral School of the University of Łódź and Łódź Institutes of the Polish Academy of Sciences, Faculty of Biology and Environmental Protection, University of Łódź, Łódź, Poland; 5 Protein Mass Spectrometry Laboratory, University of Wrocław 49572, Wrocław, Poland; 6 Department of Immunology and Infectious Biology, Faculty of Biology and Environmental Protection, University of Łódź 196812, Łódź, Poland; CNRS-University of Toulouse, Toulouse, France

**Keywords:** microbiology, mycobacterium, nucleoid-associated proteins, cell cycle, cell division, transcriptome

## Abstract

The bacterial chromosome is organized in a hierarchical and dynamic manner to facilitate essential DNA transactions. Nucleoid-associated proteins (NAPs) are abundant, small-scale chromosomal organizers that also play important roles in maintaining chromosomal DNA integrity, regulating gene expression and DNA replication, and facilitating DNA repair. Here, we show that under optimal growth conditions, NapM—a unique mycobacterial NAP—modulates the expression of nearly one-sixth of *M. smegmatis* genes, with a notable impact on pathways involved in metabolism and cell envelope biosynthesis. Furthermore, we demonstrate that in response to cell-envelope-targeting agents, NapM localizes to the septum. Moreover, its absence contributes to the lower stability of septal DivIVA, a key component of the divisome, thereby ensuring accurate cell division under stress. Together, our findings suggest that NapM supports mycobacterial cell division by modulating the transcriptomic landscape and by associating with the divisome. These functions distinguish NapM from canonical nucleoid-associated proteins and prompt a re-evaluation of its functional classification.

**IMPORTANCE:**

Understanding how bacteria adapt to environmental stress is critical to combating antibiotic resistance. This study characterizes NapM, a stress-responsive nucleoid-associated protein (NAP) in *Mycobacterium smegmatis* that couples maintenance of the transcriptional profile with the function of the cell division machinery. Distinct from canonical NAPs, NapM is essential for the proper expression of genes involved in metabolism and cell envelope biosynthesis. It also exhibits stress-dependent septal localization. This dual functionality reveals a unique adaptive strategy by which mycobacteria coordinate envelope maintenance and cytokinesis, advancing our understanding of bacterial stress resistance and identifying a potential target for therapeutic development.

## INTRODUCTION

Emerging evidence shows that the bacterial chromosome resembles eukaryotic chromatin, having a highly organized and hierarchical structure (1). Although the chromosome must be tightly condensed to fit into the bacterial cell, certain regions need to be accessible to the protein machineries involved in basic DNA transactions, such as replication, transcription, and DNA repair. Different groups of proteins contribute to maintaining the hierarchical yet dynamic nucleoid structure. Among them are topoisomerases, which modulate chromosome topology (2); condensins, which contribute to long DNA fragment organization (3); and small basic proteins called nucleoid-associated proteins (NAPs) (1). NAPs organize the chromosome more locally by introducing bends (e.g., HU and IHF homologs), bridging DNA (e.g., H-NS, Fis), bunching and wrapping (e.g., IHF, H-NS), and stiffening (e.g., HU, Fis) (4). Moreover, they can bind DNA to act as global regulators of transcription (5–7). While some NAPs exhibit structural and/or functional homology across various bacterial species, others are conserved within a single genus or species.

The genus *Mycobacterium* possesses a distinct set of NAPs with unique functions, often beyond shaping chromosome architecture (e.g., regulation of chromosome replication and gene transcription) (8–11). Mycobacteria elongate apically and divide asymmetrically, such that the chromosome is positioned closer to the new pole (11). Moreover, the structure of the mycobacterial chromosome is unusual within the bacterial world, as it has bead-like chromosomal domains, the formation of which remains unknown (12). The most abundant mycobacterial NAP (mNAP), HupB—whose N-terminal domain resembles that of bacterial HU—has been proposed to contribute to the formation of the pre-replication complex (11) and to inhibit RecA-promoted strand exchange (13). Another distinctive mycobacterial NAP is Lsr2, a functional homolog of H-NS, the major conserved NAP in bacteria (14). Lsr2 plays a dual role in large-scale chromosomal organization and in the transcriptional silencing of horizontally acquired and/or AT-rich DNA. In *M. smegmatis*, Lsr2, together with its ortholog MSMEG_1060, promotes adaptation to stress conditions by modulating the transcription of multiple genes, including those involved in lipooligosaccharides (LOS) synthesis (8, 15). Recently, Lsr2 has been shown to promote the exchange of replicative DNA polymerase during chromosomal DNA synthesis, balancing mutagenesis and survival under DNA-damaging conditions and contributing to the emergence of antibiotic (e.g., rifampicin) resistance (16). Another mNAP, mIHF, is essential for the integration of mycobacteriophage L5 (17, 18), and its depletion results in chromosome shrinkage and inhibition of DNA replication (19). Additionally, certain NAPs are encoded exclusively in the genomes of pathogenic mycobacteria, including virulence regulators, such as EspR (20) and NapA (21).

The recently identified nucleoid-associated protein, NapM, is a well-conserved protein found in both saprophytic and pathogenic species of *Mycobacterium* (22). NapM in *M. smegmatis* was shown to be involved in altering resistance to ethambutol (EMB) and rifampicin—antibiotics used to treat tuberculosis (22). Interestingly, in *M. tuberculosis*, NapM inhibits DNA replication by binding the replication initiation protein, DnaA (23). Thus, NapM may facilitate the persistence of *M. tuberculosis* as non-replicating cells within macrophages during latent tuberculosis infections (22, 23). Although *M. smegmatis* and *M. tuberculosis* belong to the same genus, their occupied niches differ significantly. *M. smegmatis* primarily inhabits water and soil environments, often living symbiotically with other organisms, such as free-living amoebae (24). However, in immunocompromised patients, *M. smegmatis* can cause opportunistic infections that mainly affect the skin and soft tissues (25). It is unclear whether the NapM in saprophytic *M. smegmatis* is involved in chromosome replication as it is in *M. tuberculosis*.

In this study, we show that NapM modulates cell cycle dynamics and the transcriptional profile by impacting over one-sixth of *M. smegmatis* genes, particularly those involved in cell envelope biosynthesis and metabolism. Moreover, we demonstrate that NapM undergoes stress-induced relocalization to the septum, a phenomenon not previously observed for other NAPs. Using pull-down and bacterial two-hybrid (BTH) assays, we show that NapM interacts with divisiome components, including a key septation regulator DivIVA (referred to as Wag31 in mycobacteria), indicating NapM’s involvement in the cell division process. These findings indicate that NapM exhibits a broader range of functions than its originally proposed role as a nucleoid-associated protein, raising questions about its classification within this group.

## RESULTS

NapM is annotated as a member of the PadR family, a large group of transcriptional regulators that function as environmental sensors. PadR family members, including NapM, possess two domains: an N-terminal DNA-binding winged helix-turn-helix (wHTH) domain and a C-terminal domain responsible for homodimerization (26). Clustering analysis of NapM protein sequences across mycobacterial species did not reveal motifs characteristic exclusively of pathogens or saprophytes (Fig. S1A). Despite the high overall sequence conservation (average identity of 92%), considerable variability was observed in the C-terminal region, which mediates protein-protein interactions (Fig. S1B). Earlier studies suggested that NapM is a DNA-bridging protein that forms aggregates with looped dsDNA *in vitro* (22, 27), but it was not demonstrated whether dimerization/oligomerization of NapM is involved in the formation of such structures. To address this, we carried out BTH experiments and found that NapM forms homodimers (Fig. S2A), and the dimer structure is presumably stabilized by interactions of the C-terminal domains, as further supported by AlphaFold predictions (Fig. S2B). Additionally, the recently solved crystal structure of NapM from *M. tuberculosis,* which reveals the formation of homodimers, further supports our results (https://doi.org/10.2210/pdb8jxk/pdb).

### NapM mediates cell elongation and cell cycle dynamics

**Fig 1:**
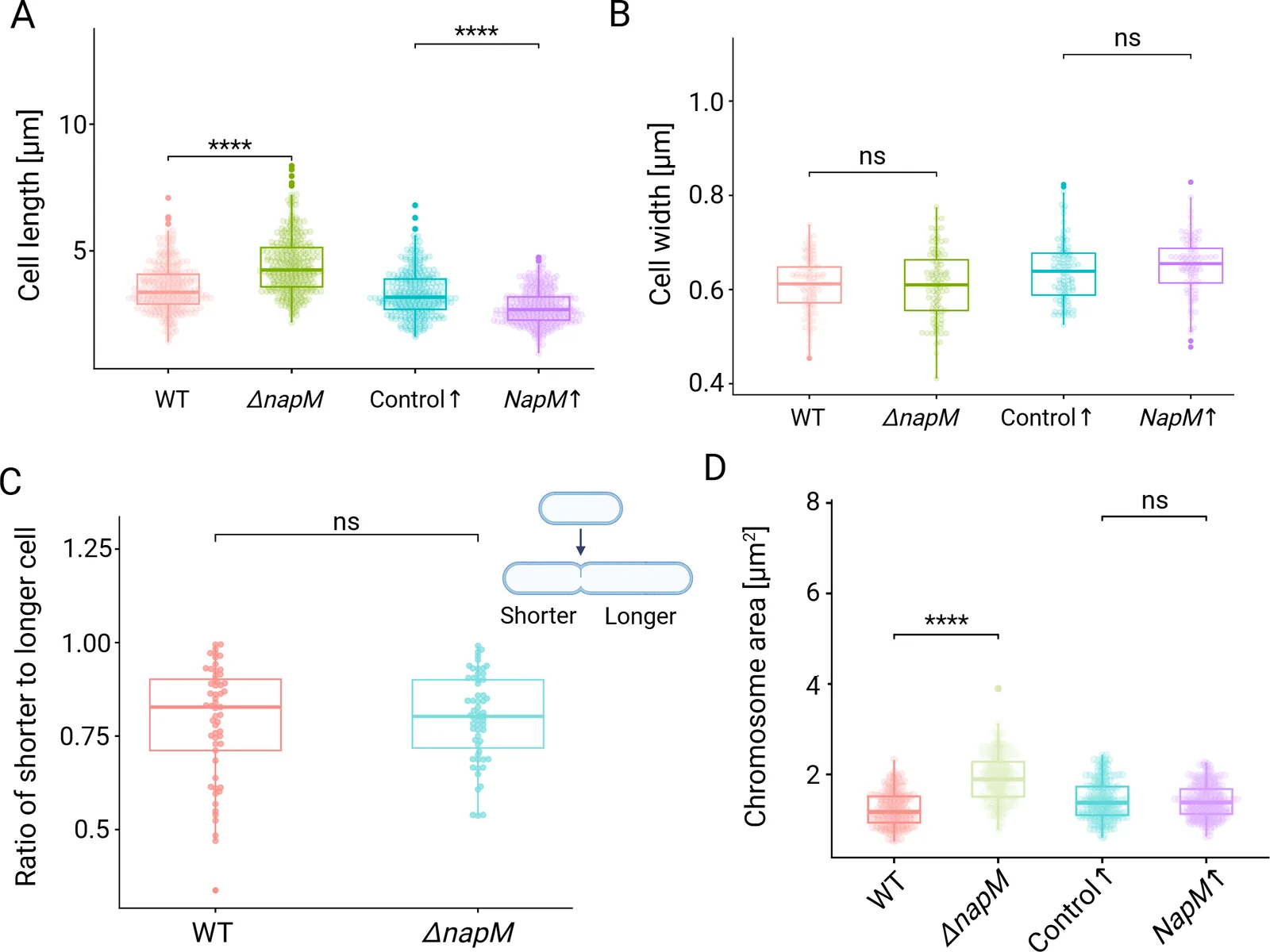
The *napM* deletion alters cell and chromosome length. Boxplots presenting the average cell lengths (**A**), cell width (**B**), daughter cells asymmetry (**C**), and area (**D**) of the chromosomes stained with Hoechst 33342 dye in the analyzed strains. WT, wild type; ∆*napM*, WT with deleted *napM*; Control↑, WT with integrated empty pMV_pAMI_ vector; NapM↑, WT with integrated pMV_pAMI_ vector containing NapM sequence under inducible promoter. Control↑ and NapM↑ were induced with 1% acetamide. ns, not significant; ****P < 0.0001.

To elucidate the function of NapM during *M. smegmatis* growth, we analyzed the single-cell morphology and chromosome organization of the *napM* deletion mutant (Δ*napM*) and a strain overproducing NapM protein (NapM↑) under optimal conditions (see Materials and Methods). The Δ*napM* cells were longer than WT cells (4.40 ± 2.10 µm and 3.50 ± 1.40 µm, respectively; t(598) = 8.43, *P* = 2 × 10⁻¹⁶, *n* = 300 per group; Fig. 1A), whereas the NapM↑ cells were shorter than Control↑ cells (WT strain with an empty pMV_pAMI_ vector after induction with 1% acetamide; 2.70 ± 0.70 µm and 3.30 ± 0.90 µm, respectively; t(598) = −9.11, *P* = 3.20 × 10⁻¹⁶, *n* = 300 per group; see Fig. 1A). The average cell width was similar across all strains (Fig. 1B), and no significant differences were observed in the asymmetry ratio of daughter cells (Fig. 1C). These data suggest that NapM may influence cell elongation and/or cell envelope composition in *M. smegmatis,* potentially through transcriptional regulation. Although no morphological abnormalities were observed in the tested strains (Fig. S3A), deletion of *napM* significantly increased nucleoid area (1.91 ± 0.52 µm^2^ versus 1.23 ± 0.38 µm^2^ in the wild type; t(325.82) = −13.98, *P* < 2.22 × 10^−16^, *n* = 169 and 179, respectively; Fig. 1D). However, nucleoid spatial organization, quantified as the percentage of cell length occupied by the chromosome, remained unchanged (Fig. S3B, *n* = 194 and 185 for ∆*napM* and WT, respectively). The preserved spatial proportion coincided with an increase in cell length following *napM* deletion, potentially contributing to the increased nucleoid area. Additionally, nucleoid area and condensation did not differ between NapM↑ cells and the Control↑ strain (Fig. 1D; Fig. S3B).

Since NapM in *M. tuberculosis* was previously linked to DNA replication (23), we used the replisome marker, DnaN-mCherry, as described by Trojanowski et al. (28), to monitor its dynamics in the WT and Δ*napM* strains during the *M. smegmatis* cell cycle. The duration of the C phase (replication time) was measured by tracking the DnaN-*mCherry* foci, which mark sites of active DNA synthesis. Conversely, the BD phase was defined as the interval between the disappearance of the foci in the mother cell and their reappearance in newly divided daughter cells, corresponding to cell division and the initiation of a new round of replication cycle (Fig. S4A). Time lapse fluorescence microscopy (TLFM) experiments revealed that, under optimal conditions, Δ*napM* cells exhibited a slight increase in replication time (C phase) compared to WT cells (126 ± 21 min and 120 ± 17 min, respectively; t(608) = 3.46, *P* = 5.4 × 10^−4^, *n* = 305 per group; see Fig. S4B). However, no significant difference was observed in the duration of the BD phase (Fig. S4B). Interestingly, under nutrient-restriction conditions (limited nutrients availability, see Materials and Methods), Δ*napM* strain exhibited prolongation of both the C phase (132 ± 18 min vs. 124 ± 1 min; t(198) = 4.14, *P* = 3.6 × 10^−6^, *n* = 272 for ∆*napM*, 267 for WT) and, particularly, the BD phase (58 ± 27 min vs. 46 ± 20 min; t(198) = 3.67, *P* = 1 × 10^−10^ *n* = 326 for ∆*napM*, and 314 for WT, see Fig. S4C). Consequently, the doubling time of the ∆*napM* strain was extended compared to the WT strain (190 ± 35 min and 171 ± 28 min, respectively; t(498) = 6.93, *P* = 2.2 × 10^−12^, *n* = 250).

These results suggest that NapM influences cell size and replisome dynamics in *M. smegmatis*. Under optimal conditions, its absence leads to cell elongation, whereas its overexpression results in cell shortening, indicating that NapM may mediate growth and/or division in *M. smegmatis*. Moreover, under nutrient-limiting conditions, the absence of NapM further prolongs replisome activity during the C phase and delays the initiation of a new replication round, implying that NapM contributes to the timely completion of chromosome replication and facilitates the adaptation of chromosome replication to changing environmental conditions.

### Loss of NapM triggers pleiotropic de-repression of genes involved in cell envelope biogenesis and cofactor biosynthesis

**Fig 2:**
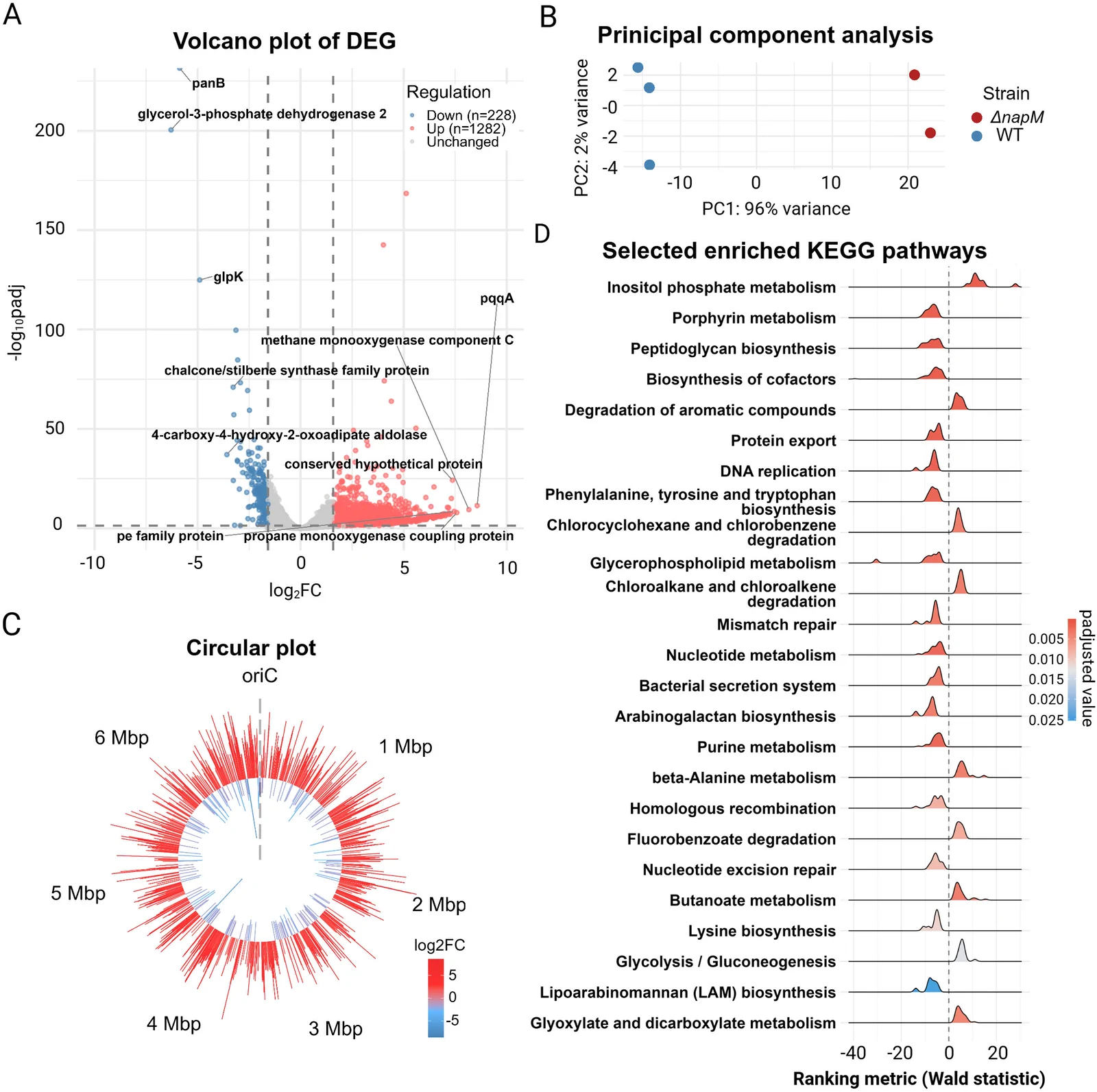
Transcriptomic analysis of *M. smegmatis* Δ*napM* deletion mutant compared to the wild-type strain (WT). (**A**) A volcano plot of DEGs (|log2FC| ≥ 1.585, *P*adj < 0.05). Genes annotated are these with the highest fold change. (**B**) Principal component analysis (PCA) of variance-stabilizing transformed (VST) counts, showing clear separation of WT and Δ*napM* replicates. (**C**) A circular genome plot showing chromosomal distribution of DEGs, including bar height and color reflecting log_2_FC. (**D**) The ridge plots of KEGG pathways, which are significantly enriched in the Δ*napM* strain, were identified by GSEA. The x-axis represents the Wald statistic distribution for genes within each pathway, positive and negative values indicate overall transcriptional upregulation or downregulation in the ∆*napM* strain, respectively. The Wald statistic (log_2_FC divided by its standard error) was used as a ranking metric, integrating both the magnitude and precision of gene expression changes. The ridge plot colors correspond to Benjamini-Hochberg adjusted *P*-values for each pathway, indicating the statistical significance of the enrichment.

Since NapM belongs to the PadR family of transcription factors, we investigated whether its deletion would affect the transcriptome. To this end, we performed RNA-seq (GEO accession GSE275852) to compare the global transcription profiles of Δ*napM* and WT strains grown under optimal conditions. Deletion of *napM* significantly altered the expression of 1,512 genes (abs log_2_FC ≥ 1.585 and *P*-value “adjusted” ≤ 0.05) in exponentially growing *M. smegmatis* cells. A log_2_FC threshold of 1.585 (threefold change) was applied to capture the widespread but moderate transcriptional changes characteristic of NAP-mediated regulation, which typically reflects alterations in global gene expression profile rather than large expression shifts at individual loci. Notably, 1,283 genes exhibited increased expression, while 229 showed decreased expression in the Δ*napM* strain, indicating that the absence of NapM predominantly leads to transcriptional de-repression (Fig. 2A; Table S1).

PCA (Prinicipial Component Analysis) was performed to assess relationships among samples and the overall variance structure. The first and second principal components accounted for 96% and 2% of the total variance, respectively. Samples clustered clearly according to strain, with biological replicates tightly grouped (Fig. 2B). The top five upregulated and downregulated genes are annotated on the volcano plot (Fig. 2A).

The highest log_2_FC value is observed for the *pqqA* gene, which encodes the rate-determining peptide required for pyrroloquinoline quinone (PQQ) synthesis. PQQ is an essential redox cofactor for dehydrogenases and can also function as an antioxidant. The most strongly downregulated transcript corresponds to *glpD*, which encodes an enzyme involved in glycerol utilization and cellular energy metabolism, reducing FAD to FADH_2_ in the respiratory chain. Among other genes showing substantial changes in expression are *panB* (downregulated), which encodes a key enzyme in vitamin B_5_ biosynthesis (29, 30); *glpK* (downregulated), which encodes the glycerol kinase that activates glycerol as a substrate for downstream enzymes such as GlpD; MSMEG_1972 (upregulated), which encodes a subunit of a monooxygenase complex responsible for transferring electrons from NADH or NADPH to complex; and *ino1* (upregulated), which encodes the enzyme catalyzing the first step of *de novo* myo-inositol synthesis (Table S1). Notably, *ino1*-deficient cells require exogenous inositol for growth (31).

Differentially expressed genes (DEGs) were broadly distributed across the entire chromosome (Fig. 2C) with no apparent positional bias. To identify the metabolic pathways affected by the loss of *napM*, we performed KEGG pathway enrichment analysis using a gene set enrichment analysis (GSEA) approach (32) (Fig. 2D; Table S2). This method enables the detection of pathway-level transcriptional changes that might be overlooked when applying a strict threshold for single-gene differential expression. By evaluating genes within their broader pathway context, GSEA captures coordinated but modest alterations that may be as biologically relevant as large individual effects.

Interestingly, although the majority of DEGs were upregulated, *napM* deletion largely resulted in the downregulation of enriched pathways. These included cofactor biosynthesis, porphyrin metabolism, and notably, peptidoglycan (PG) biosynthesis. The most strongly de-repressed pathway was inositol phosphate metabolism, which includes the previously mentioned *ino1* gene. Additional upregulated pathways included the degradation of aromatic compounds, xenobiotics, and fluorobenzoate. Several enriched pathways were consistent with the observed ∆*napM* phenotype of elongated cells. These included inositol phosphate metabolism, peptidoglycan biosynthesis, DNA replication, glycerophospholipid metabolism, and arabinogalactan and lipoarabinomannan (LAM) biosynthesis. The full list of enriched pathways, along with the associated genes, is provided in Table S2. Finally, we also examined individual genes not annotated within KEGG pathways but previously known to be associated with cell elongation or cell division (e.g., *divIVA, crgA, MSMEG_6171*) and cell envelope synthesis. These and other relevant genes are listed in Table S3, including those that did not meet the single-gene log_2_FC threshold (Table S1) but are known to participate in pathways identified as significant via GSEA (Fig. 2D; Table S2).

Overall, these results demonstrate that the absence of NapM triggers pleiotropic transcriptional reprogramming. The affected transcripts of pathways converge on key processes such as catabolism, DNA replication, and cell envelope biogenesis, consistent with the phenotypic alterations observed in the ∆*napM* strain.

### Absence of *napM* disrupts PIM homeostasis, resulting in the accumulation of PIM_2_, a building block of the cell envelope

Deletion of *napM* caused an extended cell phenotype but also resulted in transcriptional repression of cell envelope biosynthesis (Fig. 2D; Table S2). For instance, PG biosynthesis genes were downregulated across all synthesis steps, including cytoplasmic precursor synthesis, the formation and translocation of lipid-linked intermediates across the cytoplasmic membrane, and PG polymerization and cross-linking (Table S3). Additionally, lipoarabinomannan biosynthesis pathways were transcriptionally repressed in the ∆*napM* strain, with several genes involved in phosphatidylinositol-containing polar lipid biosynthesis (Fig. 2D). These genes span multiple steps, from phosphatidylinositol (PI) synthesis through phosphatidylinositol mannosides (PIMs) formation to their glycosylation into lipomannan (LM) and lipoarabinomannan (LAM) (Table S3).

**Fig 3:**
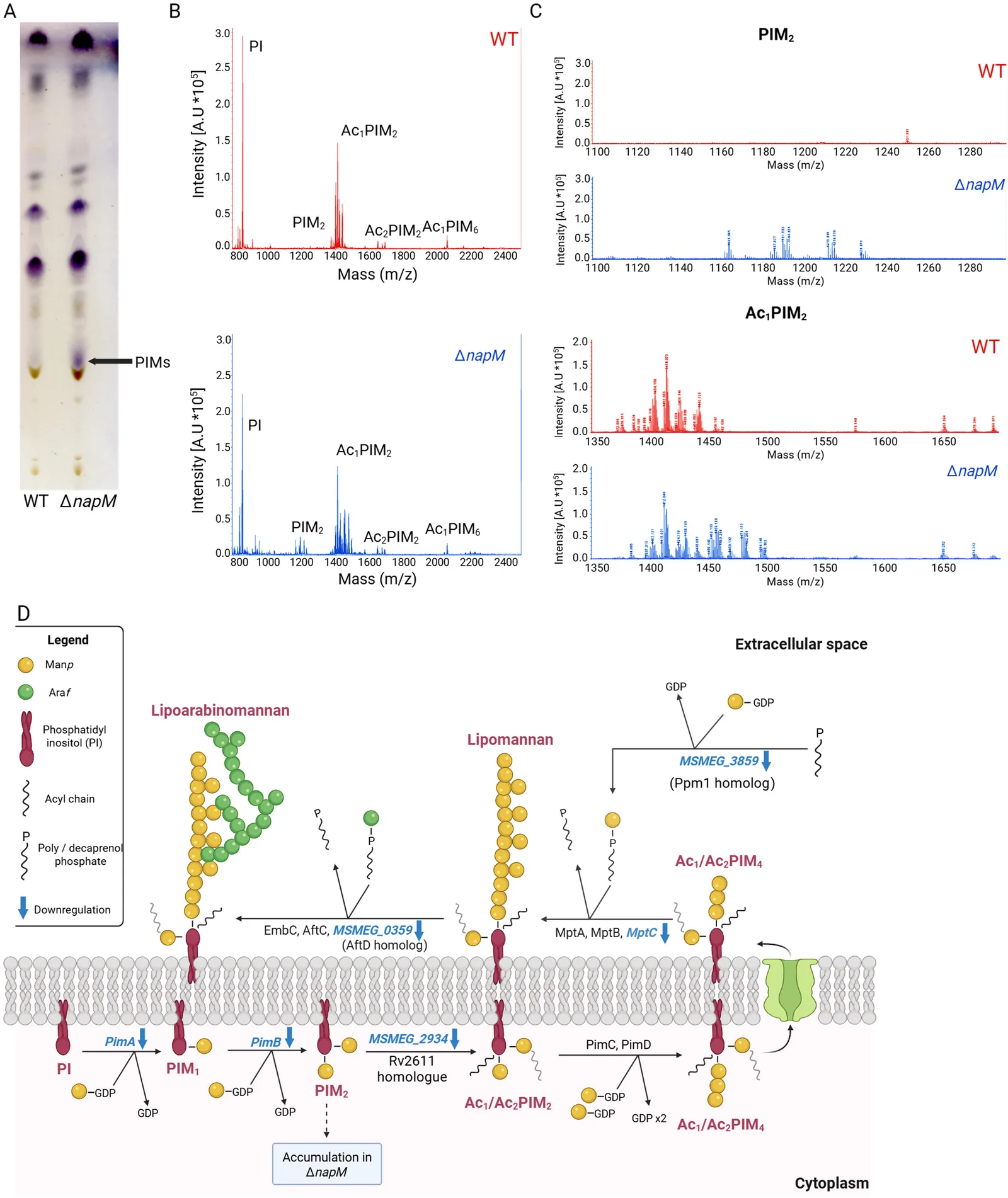
The *napM* deletion results in altered PIM composition. (**A**) TLC analysis of the total lipid fractions of the WT and ∆*napM* strains shows enrichment in the PIM fraction. (**B**) MALDI-TOF MS analysis of lipids in the WT and ∆*napM* strains. PIM_2_ is being accumulated in ∆*napM* (blue) strain, and the acylated PIM_2_ level is lowered in comparison to the WT strain (red). (**C**) Zoomed regions of MALDI-TOF MS spectra of PIM_2_ (phosphatidylinositol dimannoside) and Ac_1_PIM_2_ (acylated phosphatidylinositol dimannoside) obtained from the WT (red) and ∆*napM* (blue) cells. (**D**) Schematic biosynthesis pathway, with implemented transcriptomic changes of (blue—downregulated; red—upregulated) polar lipids (PI, LM, and LAM) in *M. smegmatis* ∆*napM* strain.

These metabolically related polar lipids share a conserved glycosylated phosphatidylinositol (GPI) anchor and are integral components of the mycobacterial cell envelope. Disruptions in their synthesis increase bacterial susceptibility to stress (e.g., ethambutol treatment) while also affecting cell shape and division (33). Since RNA-seq results suggested potential alterations in mycobacterial cell envelope lipid composition, we performed thin-layer chromatography (TLC) of lipid extracts, which revealed an enrichment of the PIMs fraction in the Δ*napM* strain compared to the WT strain (Fig. 3A). Additionally, the observed downregulation within the PG synthesis pathway, and to a lesser extent in the arabinogalactan (AG) synthesis pathway (Fig. 2D; Table S2), suggests a disruption of the cell wall skeleton structure which is crucial for the integrity of the mycolic acid (MA) layer anchored in the PG-AG matrix (34). To further investigate cell wall lipid composition, we performed TLC of mycolic acid methyl esters (MAMES) (Fig. S5A). However, no significant changes were observed.

Further analysis of total lipids from the Δ*napM* strain using 2D-TLC (Fig. S5B and C) confirmed the presence of an additional, novel lipid species, including phospholipids. Subsequent MALDI-TOF MS analysis revealed an enrichment of non-acylated PIM_2_ (phosphatidylinositol dimannoside) (Fig. 3B and C). The increase in PIM2 levels in the ∆*napM* strain is described qualitatively, as MALDI-TOF data are inherently semi-quantitative and are consistent with our biochemical and phenotypic observations (Fig. 1 and 3; Fig. S5). Based on the co-occurrence of the additional 2D-TLC spot and the PIM_2_ enrichment detected by MS, we hypothesize that PIM_2_ constitutes the major component of these lipids. A similar PIM profile, characterized by the accumulation of PIM_2_ and a reduction in its acylated form (Ac_1_PIM_2_), was previously observed in a *M. smegmatis* strain lacking *MSMEG_2934*, the gene encoding the acyltransferase responsible for adding an acyl group to PIM_2_ (31). This observation is consistent with our RNA-seq results, which show that in the Δ*napM* strain, *MSMEG_2934* is the most downregulated gene (log_2_FC = −1.71) in the PIMs biosynthesis pathway (Fig. 2D and 3D; Table S3). Moreover GPI metabolism can be impacted by via exogenous inositol uptake, which transcripts are elevated in our RNA-Seq (Figure 2; Table S1; Fig. S6).

Additionally, to explore whether NapM directly interacts with enzymes involved in cell envelope biogenesis, we performed a pull-down experiment using NapM fused with a FLAGx3 tag. Subsequent mass spectrometry analysis revealed that no proteins from the LAM synthesis pathways were detected in the NapM interactome (Fig. S7; Table S4). Instead, NapM copurified with the divisome-associated proteins CwsA, SepIVA—key regulators of divisome and KhpA, which has been shown in *S. pneumoniae* to play a role in cell shape and division (35–38).

Our findings indicate that the absence of NapM impairs cell envelope homeostasis in *M. smegmatis*, as evidenced by PIM_2_ accumulation. Although direct interactions between NapM and members of LAM biosynthesis pathway were not detected, the NapM interactome includes the divisome regulators CwsA, SepIVA, and KhpA.

### NapM affects cell resistance to stress

**Fig 4:**
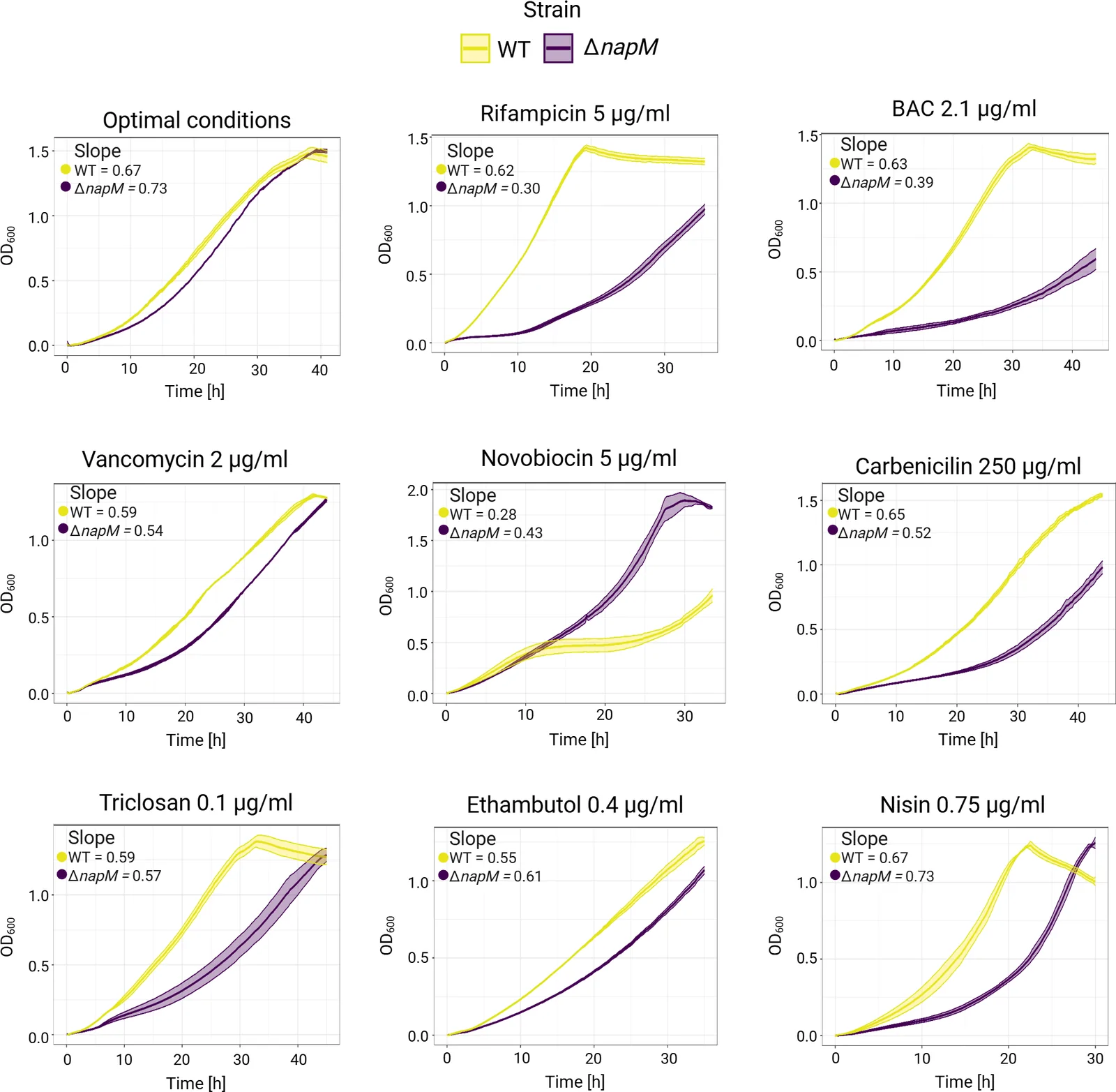
Loss of *napM* affects the growth of *M. smegmatis* under various stress conditions. Growth curves of the Δ*napM* strain compared to the wild-type *M. smegmatis* mc^2^ 155 (WT) strain.

Our previous RNA-seq experiments (15) suggest that the *napM* gene is expressed at a relatively low level in exponentially growing *M. smegmatis* cells. Additionally, data from the protein abundance database (PaxDb, https://pax-db.org/protein/246196/MSMEG_6903) indicate that NapM protein constitutes a minor fraction of the total cellular proteome, with levels between 12.0 and 17.7 ppm, compared to 12,000–14,000 ppm for HupB. Intriguingly, the *napM* transcript level in pathogenic *M. tuberculosis* increases upon exposure to various stresses, including environmental factors and antibiotics (23). A similar phenomenon was observed in our previous RNA-seq experiment, where *napM* transcript levels in *M. smegmatis* increased under oxygen depletion conditions (15). Given these findings, we decided to investigate the impact of NapM absence on *M. smegmatis* growth in response to stress, specifically in the presence of antimicrobial compounds. We examined the growth of the Δ*napM* strain under stress conditions induced by disinfectants (general environmental stressors) and antibiotics targeting different cellular processes, including cell envelope biosynthesis, transcription, and replication (Fig. 4). The Δ*napM* strain exhibited a prolonged adaptation phase and reduced growth compared to the WT strain when exposed to agents that compromise cell envelope integrity through distinct mechanisms. The most pronounced growth defects were observed with benzyldodecyldimethylammonium chloride (DDBAC; referred to throughout the manuscript as BAC), which disrupts the cytoplasmic membrane via surfactant-mediated damage, followed by carbenicillin, a β-lactam antibiotic that targets penicillin-binding proteins (PBP) transpeptidases and transglycosylase essential for PG crosslinking. Growth of the Δ*napM* strain was also impaired in the presence of nisin, which binds lipid II and forms membrane pores, and triclosan, an inhibitor of fatty acid biosynthesis, indirectly destabilizing membrane structure. More modest growth defects were detected with ethambutol, which blocks arabinosyltransferases (EmbA–C) involved in arabinogalactan and lipoarabinomannan synthesis, and vancomycin, which interferes with peptidoglycan cross-linking by binding to the D-Ala-D-Ala termini of peptidoglycan precursor. In the NapM↑ strain, complete growth inhibition was observed only in the presence of vancomycin and nisin (see Fig. S8). As the Δ*napM* strain likewise exhibited reduced growth under these conditions (Fig. 4), our findings indicate phenotypic convergence arising from disruption of transcriptional homeostasis following either NapM depletion or overproduction.

In the presence of rifampicin, a transcription inhibitor, Δ*napM* cells showed significantly reduced growth. Unexpectedly, treatment with novobiocin, a gyrase B subunit inhibitor, resulted in enhanced growth of the mutant strain (Fig. 4), suggesting a NapM-mediated bypass mechanism that helps maintain proper chromosome DNA topology under treatment with this antibiotic. In our RNA-seq experiments, no changes were detected in the transcripts of *gyrB* (MSMEG_0005) or *MSMEG_0457* (encoding the DNA topoisomerase IV subunit B), but the upregulation of *MSMEG_1229*, annotated as a homolog of *gyrB* (which lacks in *M. tuberculosis*), may compensate for the novobiocin action.

To further investigate NapM dynamics, we analyzed NapM-FLAG levels via Western blot in response to selected stress agents. We observed an increase in NapM-FLAG levels upon exposure to the cell-envelope-disrupting agents carbenicillin and ethambutol (Fig. S9). Although these changes did not reach statistical significance, potentially due to the inherent variability of semi-quantitative immunoblotting at a standard sample size (*n* = 3), the observed biological trend is consistent with the impaired growth of the ∆*napM* strain under these conditions (Fig. 4). In contrast, exposure to rifampicin resulted in a modest reduction in NapM-FLAG levels, suggesting that NapM regulation may differ under stresses imposed by antibiotics that do not share the same molecular target.

Together, these results suggest that NapM levels are modulated under stress conditions and that NapM contributes to *M. smegmatis* fitness, particularly during exposure to cell envelope-targeting agents.

### NapM localizes at the septum in response to stress

**Fig 5:**
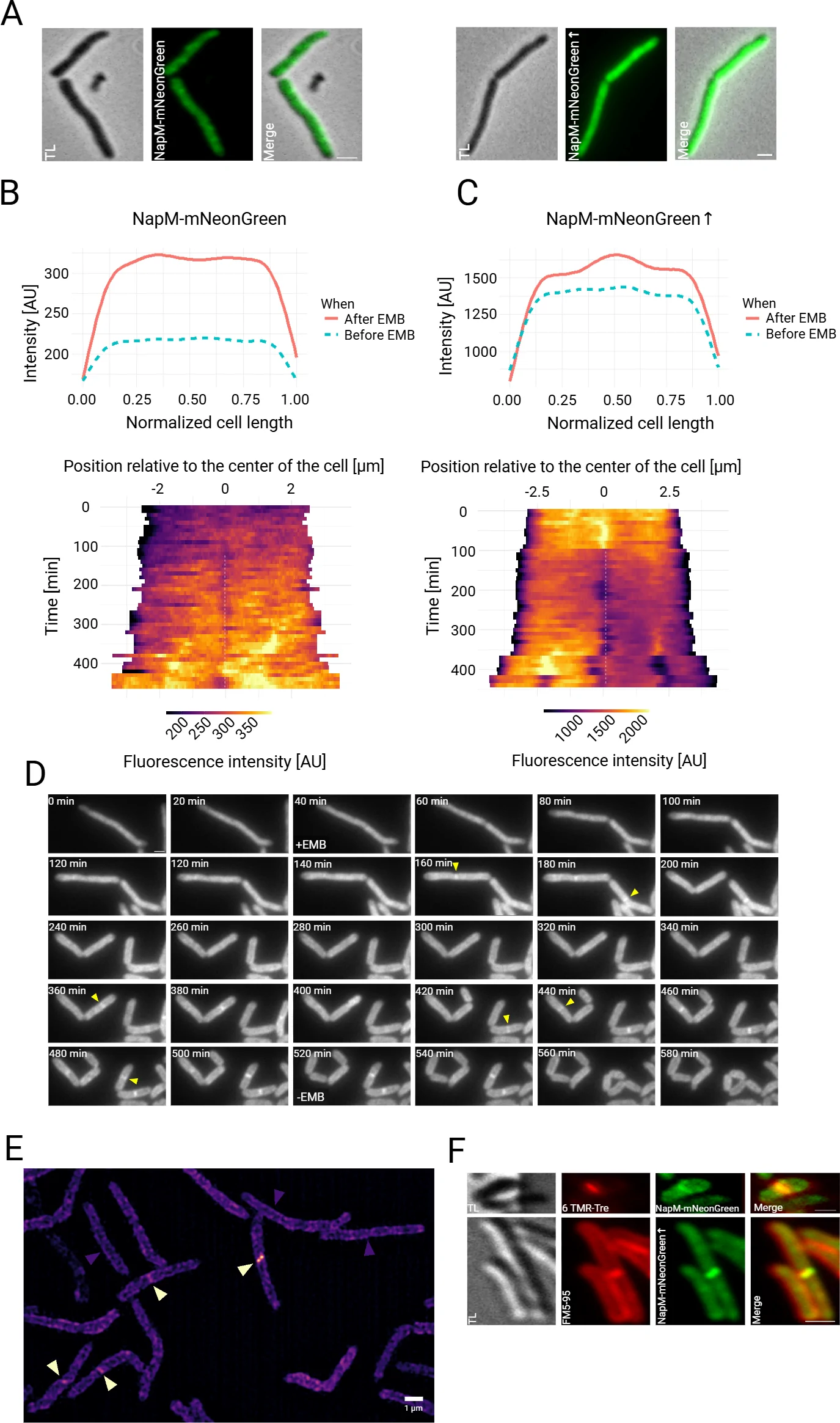
NapM localizes at the septum after treatment with ethambutol (EMB). (**A**) NapM-mNeonGreen localization under optimal conditions. Averaged fluorescence profile measured along the long axis of the cell (*n* = 100) and kymograph for a representative cell expressing NapM-mNeonGreen from the native locus (**B**) and for cells overexpressing NapM-mNeonGreen (NapM–mNeonGreen↑) (**C**). (**D**) Time-lapse analysis of the *M. smegmatis* NapM-mNeonGreen↑ strain, showing localization of NapM-mNeonGreen at the septum area (yellow rectangles) after exposure to EMB. (**E**) Representative high-resolution Lattice-SIM image of NapM-mNeonGreen*↑* cells following EMB treatment. Cream-colored arrowheads indicate septal localization of NapM-mNeonGreen. Image was processed in ImageJ using an alternative LUT (mpl-inferno). Scale bar: 1 µm. (**F**) NapM-mNeonGreen and NapM-mNeonGreen*↑* cells stained, respectively, with cytokinesis marker, FM5-95 or TMR Tre (fluorescently labeled trehalose, cell envelope component) showing colocalization of NapM-mNeonGreen complexes with the formed septum after EMB treatment. Scale bar, 1 µm. TL, transmitted light.

In light of the association between NapM and cell survival during exposure to cell envelope–targeting agents, conditions that also correlate with the tendency of NapM to accumulate, we examined NapM subcellular localization under both optimal and stress conditions. Previous studies demonstrated that NapM colocalizes with chromosomal DNA in *E. coli* cells (22), a finding we confirmed in our experiments (Fig. S10). Consistent with this, NapM from *M. smegmatis* binds DNA *in vitro* (22) and NapM in *M. tuberculosis* displays extensive genome-wide binding, with 573 sites identified by Chip-Seq analysis (38). To analyze the localization of NapM during the *M. smegmatis* cell cycle, we constructed a strain in which *napM* was fused to the *mNeonGreen* gene at its native locus (subsequently referred to as NapM-mNeonGreen strain). Fluorescence microscopy analysis of NapM-mNeonGreen cells under optimal conditions revealed a diffuse distribution of the fusion protein, with no distinct localization (Fig. 5A, left panel).

Because the fluorescence signal from the native NapM-mNeonGren fusion was low under optimal conditions—likely reflecting low native *napM* expression—and because NapM overexpression had minimal impact on growth (Fig. S8), we constructed a merodiploid strain containing an additional *napM-mNeonGreen* fusion gene under the control of an inducible promoter (subsequently referred to as NapM-mNeonGreen↑ strain), while retaining *napM-mNeonGreen* at its native locus. This approach increased the signal-to-noise ratio and allowed us to determine whether elevated NapM levels (Fig. S11 and S12) reveal specific localization under optimal conditions or whether the signal is enhanced and stabilized during stress. Although the NapM-mNeonGreen↑ strain indeed displayed a stronger fluorescence signal compared to the native NapM-mNeonGreen strain, similarly to the previous strain, no distinct localization patterns were observed under optimal conditions (Fig. 5A, right panel). However, upon ethambutol treatment, fluorescence intensity markedly increased in both strains (Fig. 5B and C), and robust septal localization of NapM-mNeonGreen was observed in many of the dividing cells of the merodiploid strain (Fig. 5D and E; Movies S1 and S2). For the native-expression strain, where the signal-to-noise ratio was limited, we applied the smart LUT to enhance visual discrimination without altering signal intensity; this method was used consistently in subsequent analyses. Using this approach, population-wide septal localization was confirmed in the NapM-mNeonGreen strain following EMB treatment (Movie S1). Elevated levels in the merodiploid strain produced more sharply defined septal signals, consistent with improved signal-to-noise ratio. Variability in septal signal strength in the native strain is likely attributable to known cell-to-cell heterogeneity in mycobacterial responses to stress (39). Septal localization was also observed following exposure to triclosan (Fig. S13).

The NapM-mNeonGreen fusion protein was observed at the septum for approximately 40–50 minutes (40 ± 20 min for NapM-mNeonGreen and 50 ± 23 min for NapM-mNeonGreen↑, t(185) = −4.89, *P* = 2.14 × 10^−6^, *n* = 87 for NapM-mNeonGreen and 100 for NapM-mNeonGreen↑, Fig. S14A) and disappeared immediately after division (i.e., V-snapping) (Movies S1 and S2). Using high-resolution microscopy (Lattice SIM), we showed that NapM-mNeonGreen localizes to the septum during its formation following EMB treatment (Fig. 5E). Additional experiments using FM5-95 dye (a cytokinesis marker) and TMR-Trehalose dye (cell envelope marker) confirmed that NapM-mNeonGreen indeed colocalizes with the newly formed septum in NapM-mNeonGreen↑ and NapM-mNeonGreen strains, respectively (Fig. 5F). Analysis of cells just before division (V-snapping) revealed that cells displaying a septal NapM-mNeonGreen signal were shorter than those with dispersed cytoplasmic signal (2.85 ± 0.43 µm vs. 3.49 ± 0.72 µm; t(168) = −7.56, *P* = 4.00 × 10⁻^14^, *n* = 100 per group; see Fig. S14B). Moreover, the elongation rate was slightly elevated in cells exhibiting NapM-mNeonGreen relocalizing to septum (0.04 ± 0.06 µm/min vs 0.08 ± 0.07 µm/min; t(94) = −3.11, *P* = 2.5 × 10^−3^, *n* = 49 and 53, respectively, for cytoplasmic and septal).

Taken together, these results demonstrate that the subcellular localization of NapM differs from that of previously characterized NAPs (e.g., HupB, Lsr2, MSMEG_1060) in *M. smegmatis*. Under optimal conditions, NapM-mNeonGreen exhibits a diffuse distribution in the cell, without discrete fluorescent foci. Intriguingly, upon exposure to cell envelope-targeting drugs such as ethambutol and triclosan, NapM localizes within the septum area. This provides further evidence, alongside RNA-seq and pull-down results, that NapM plays a role in cell division.

### Loss of NapM impairs DivIVA septal localization upon ethambutol treatment

**Fig 6:**
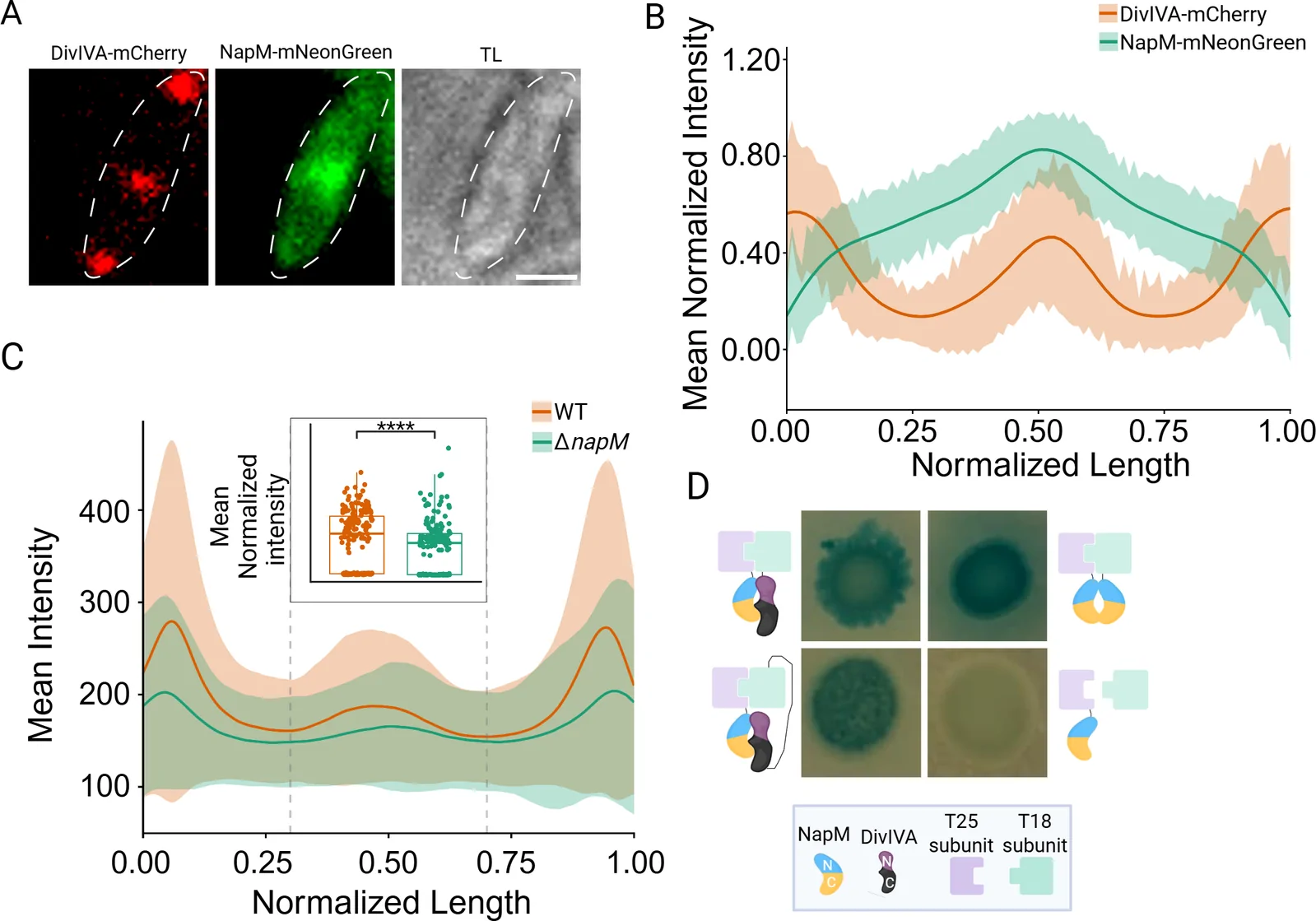
NapM colocalizes at the septum and interacts with DivIVA. (**A**) Microscopic images showing colocalization of NapM-mNeonGreen and DivIVA-mCherry at the septum. Scale bar, 1 µm. (**B**) Average fluorescence intensity profiles of DivIVA-mCherry and NapM-mNG along the long cell axis (*n* = 133). (**C**) Average fluorescence intensity profile of DivIVA-mCherry in WT and Δ*napM* strains (*n* = 173 and 171, respectively). Dashed lines mark the analyzed septal peak region (0.30–0.70). The Δ*napM* mutant exhibits a significant reduction in mean fluorescence intensity within this region. (**D**) Interaction between NapM and DivIVA was analyzed using the BTH assay. NapM homodimerization (NapM-NapM) served as a positive control (top right), and NapM with an empty vector served as a negative control (bottom right). ****P < 0.0001.

Given the unexpected septal localization of NapM-mNeonGreen under stress conditions, the dependence of cell length on NapM, the impaired PIM homeostasis, and the observed transcriptional changes in genes involved in cell envelope biosynthesis upon *napM* deletion, we sought to determine whether NapM colocalizes with DivIVA, the main regulator of divisiome (40, 41). To this end, we constructed a strain producing NapM-mNeonGreen and DivIVA-mCherry fusion proteins (see Materials and Methods section). Our microscopic observations of cells treated with EMB confirmed that NapM-mNeonGreen and DivIVA-mCherry colocalize at the newly forming septum, where DivIVA (Fig. 6A and B; Movie S3) appears approximately 64 ± 57 min (*n* = 57) before NapM. Upon V-snapping, both the NapM and DivIVA signals disappeared. To assess whether the absence of NapM affects DivIVA dynamics under EMB treatment, its localization was examined in the Δ*napM* background. To determine the impact of NapM deficiency on DivIVA-mCherry intensity and distribution, the average DivIVA-mCherry signal was analyzed across the cell, specifically within the region defined as 0.30–0.70 of the normalized cell length. This analysis revealed a lower signal intensity in the septal region of the ∆*napM* mutant compared to the wild-type strain (156.88 ± 53.57 vs 172.16 ± 60.89, *P* = 2.2 × 10^−6^, *n* = 171 and 173, respectively) (Fig. 6C). Furthermore, the overall DivIVA-mCherry intensity was slightly reduced in the absence of NapM. This observation suggested a potential NapM-DivIVA interaction, which was subsequently supported using the BTH system (Fig. 6D) and its potential interaction was visualized via AlphaFold3 (Fig. S15)

## DISCUSSION

NapM, a PadR-family transcriptional regulator, is a highly conserved protein within the *Mycobacterium* genus (Fig. S1) and forms homodimers (Fig. S2A), which is consistent with the recently resolved crystal structure of *M. tuberculosis* NapM (https://doi.org/10.2210/pdb8JXK/pdb). Both its absence and overexpression prolonged the lag phase under optimal conditions, with growth defects further exacerbated under stress (Fig. 4; Fig. S8). Compared to the abundant NAPs, such as HupB or mIHF, the NapM level is approximately 800-fold lower (PaxDB, https://pax-db.org/protein/246196/MSMEG_6903), indicating that it might primarily function as a regulatory factor rather than a structural component of the chromosome. Our findings support a role for NapM in bacterial adaptation. Deletion of *napM* impaired growth under multiple stress conditions (Fig. 4; Fig. S8), and NapM levels tended to increase in response to cell envelope-targeting agents—nisin, carbenicillin, and most prominently ethambutol (Fig. S9). Strikingly, overexpression of NapM rendered cells highly sensitive to the high-molecular-weight antibiotics vancomycin and nisin, both of which target lipid II. We propose that this phenotype arises from a combination of impaired cell envelope integrity and interference with the divisome function caused by excess NapM accumulation at the septum. Although direct evidence for this mechanism is not yet available, it is consistent with our transcriptomic, localization, and antibiotic susceptibility data. Elucidating the contribution of NapM to lipid II utilization and cell division will be an important focus of future studies.

Loss of NapM directly or indirectly affected the expression of nearly one-sixth of all genes in *M. smegmatis*. The affected pathways, together with copurified proteins, could be grouped into two major functional categories: central metabolism and cell envelope biogenesis (Fig. 2; Tables S1, S2, and S4). As the present study focused primarily on the latter, NapM’s potential involvement in central metabolic processes remains to be explored. Such extensive transcriptional perturbations are likely to have broad physiological consequences and may influence multiple stages of the cell cycle, including DNA replication. Indeed, changes in replisome dynamics were particularly evident under nutrient-limiting conditions (Fig. S4).

Unlike in *M. tuberculosis*, NapM in *M. smegmatis* does not appear to regulate replisome assembly at the initiation stage (Fig. S4). It likely does not interact with DnaA, as the protein was absent from the pull-down assay (Fig. S7; Table S4), possibly due to some differences in the C-terminal domain composition of NapM, whose sequence shows considerable diversity (Fig. S1B). The transcript level of *dnaN*, encoding the β subunit of DNA polymerase III, was lower in the Δ*napM* strain (Table S1), and consistent with this, DnaN protein levels were also reduced compared with the control (Fig. S4D). Given its essential role in the replisome, such changes in DnaN abundance may modulate DNA replication in a context-dependent manner (Fig. S4D). This observation suggests that NapM may modulate DnaN levels, thereby affecting replisome activity and potentially impacting both the C and B/D phases of the cell cycle, particularly under stress conditions. Such a response may resemble the replication inhibition described in *M. tuberculosis* (23), which has been proposed to promote survival under stress through the regulated slowdown of cell cycle progression. This could explain the reduced survival of the *napM* mutant in macrophages and its impaired growth under stressful conditions in *M. tuberculosis* and *M. smegmatis,* respectively (Fig. 4; Fig. S4). Although the precise role of NapM in regulating DNA replication in *M. smegmatis* remains to be defined, and pleiotropic effects of *napM* deletion cannot be excluded, our findings highlight a potential functional link between NapM and replisome activity that warrants further investigation.

The most pronounced changes in gene transcription were observed for *pqqA*, which was upregulated and encodes a key enzyme in pyrroloquinoline quinone (PQQ) synthesis, and *glpD*, which was downregulated and encodes a central enzyme in the respiratory chain and glycerol metabolism. While single-gene analyses highlight the most dramatic transcriptional changes (Fig. 2A), such an approach lacks pathway-level context and risks overlooking subtle but coordinated effects, particularly in the case of global transcriptional regulators or NAPs (42). Indeed, modest but concerted changes in transcript abundance across an entire pathway can be biologically significant and, in some cases, may exert a greater impact than large fold changes in individual genes (32). The GSEA (Gene Set Enrichment Analysis) revealed that the absence of NapM resulted in coordinated transcriptional downregulation of pathways involved in cell envelope synthesis. In particular, pathways associated with the synthesis of polar lipids such as PI, PIM, LM, and LAM were significantly affected in the ∆*napM* mutant (Fig. 2D; Tables S2 and S3). These lipids are core constituents of the mycobacterial cell envelope and are essential for maintaining cell wall integrity during division (33). Consistent with the transcriptomic data, lipid profiling using TLC and MALDI-TOF MS revealed an accumulation of PIMs, especially PIM_2_, in the Δ*napM* strain compared to the WT strain (Fig. 3A through C). Additionally, 2D-TLC analysis detected novel lipid species in the ∆*napM* strain (Fig. S5). These alterations are consistent with the downregulation of *MSMEG_2934*, which encodes an acyltransferase homologous to Rv2611c in *M. tuberculosis*. This enzyme catalyzes the acetylation of PIM_2_ to form Ac_1_PIM_2_/Ac_2_PIM_2_, which serve as precursors for the synthesis of LM and LAM (31, 43). Notably, deletion of *MSMEG_2934* in the WT strain produced a lipid profile (31) similar to that observed in the Δ*napM* strain (Fig. 3B), further supporting a link between NapM and regulation of polar lipid synthesis. Interestingly, although GSEA also indicated alterations in the mycolic acid biosynthesis pathway (Table S2), TLC analysis of mycolic acid methyl esters (MAMES) revealed no differences between the strains (Fig. S5). This suggests that the mycolic acid biosynthesis machinery may possess a higher tolerance for transcriptional fluctuations compared to polar lipid pathways or is buffered by robust compensatory mechanisms that appear less effective in the context of PIM metabolism.

In addition to the downregulation of genes involved in polar lipid synthesis, genes associated with PG synthesis and cell division were also transcriptionally repressed in the Δ*napM* strain (Fig. 2D; Tables S2 and S3). Consistent with these transcriptional changes, growth analysis revealed that the ∆*napM* strain exhibited reduced growth in the presence of cell-envelope-targeting agents. In particular, the increased sensitivity to carbenicillin, which inhibits PG cross-linking, is consistent with the downregulation of PG synthesis genes and suggests that the PG synthesis machinery is compromised, thereby rendering cells more susceptible to inhibition of PBP.

Analysis of the NapM interactome (Table S4) identified SepIVA and CwsA, key components involved in polar growth, cell division, and cell shape maintenance (35, 37). The observed dysregulation of cell envelope-related genes, together with these interaction partners, likely accounts for the elongated cell morphology observed under optimal conditions (Fig. 1; Fig. S3A) and the growth defects of Δ*napM* cells exposed to cell envelope-targeting stressors (Fig. 4). The pull-down assay also identified additional protein partners that may provide insight into NapM-mediated transcriptional regulation, including the ω subunit of DNA-directed RNA polymerase, which remains poorly characterized in mycobacteria. The RNA-binding protein, KhpA, was also detected among copurified proteins. Although its function has not been studied in *Mycobacterium*, KphA forms a complex with KhpB in other bacteria and participates in translational regulation of cell division genes by binding their mRNA and modulating transcript availability for translation. Loss of KhpA results in shorter and wider cells (36). Notably, these potential interaction partners, SepIVA and KhpA (Table S4), were recently co-purified in a DivIVA Co-IP experiment, and both proteins localize to the intracellular membrane domain (IMD), a proposed site of lipid synthesis in mycobacteria (44).

Despite its initial classification as a NAP, NapM did not exhibit distinct nucleoid localization under optimal growth conditions in *M. smegmatis* cells (Fig. 5A) and did not affect the nucleoid-to-cell length ratio in Δ*napM*, nor NapM↑ cells (Fig. S3B). However, the nucleoid occupied a larger area in Δ*napM* cells (Fig. 1D). This phenotype may result from reduced molecular crowding and weaker depletion forces in elongated cells. This diffused localization under optimal conditions, coupled with its profound impact on the transcriptome (Fig. 2), suggests that NapM may function as a dynamic regulatory factor rather than a tethered DNA-binding protein. We hypothesize that, in its non-septal mode, NapM could modulate gene transcription either directly or via interactions with the transcription machinery or RNA-binding partners, such as KhpA protein. The cytoplasmic pool of NapM may also serve as a reservoir, ready for rapid sequestration at the septum. Indeed, upon exposure to ethambutol (Fig. 5C through E) or triclosan (Fig. S13), NapM-mNeonGreen fluorescent signal increased and the protein displayed septal localization shortly before division (Movies S1 and S2)—a phenomenon not reported for any other NAP studied to date. While we observed a tendency for NapM to accumulate in response to multiple cell-envelope-targeting agents (Fig. S9), the distinct septal recruitment (Fig. 5D) suggests that NapM primarily responds to envelope stress through changes in subcellular localization rather than increased protein abundance alone. Consistent with this interpretation, artificial elevation of NapM levels under optimal conditions did not alter its localization. Further studies will be required to determine whether post-translational modifications or stress-specific interactions contribute to NapM relocalization and function.

Given its stress-induced septal localization (Fig. 5), interactome (Table S4; Fig. S7), and transcriptional dysregulation of cell envelope-associated pathways in the Δ*napM* strain (Fig. 3D; Tables S2 and S3), we propose that NapM influences cell division through a dual mechanism. In addition to modulating the expression of cell envelope-related genes, NapM may directly or indirectly affect the activity of the key division regulator DivIVA through interactions with proteins such as CwsA, SepIVA, particularly under stress conditions. Upon exposure to the cell envelope-targeting agent ethambutol, NapM relocalized to the septum, where it colocalized with DivIVA at the division site. Using the BTH system, we confirmed an interaction between NapM and DivIVA proteins (Fig. 6D). Consistently, ∆*napM* cells displayed a slightly more diffuse DivIVA-mCherry signal at the septum under ethambutol stress, and overall intensity was reduced despite the same imaging conditions. This observation aligns with RNA-seq data that show lower *divIVA* transcript levels compared with the wild-type strain (Fig. 2; Table S3), suggesting that NapM contributes directly or indirectly to DivIVA abundance and its organization/stabilization during cell division. NapM may ensure that, under stress conditions, cells maintain sufficient and dynamically distributed DivIVA levels, particularly at the septum, which is more prone to depletion than the polar regions. Importantly, NapM did not colocalize with the polar DivIVA pool, likely reflecting distinct functional contexts of the stable polar scaffold and the transient divisome complex. We therefore propose that NapM preserves divisome integrity through two complementary mechanisms: maintaining sufficient intracellular DivIVA levels by sustaining *divIVA* transcript abundance (Tables S1; S3), and stabilizing the division machinery via stress-induced septal localization (Fig. 5) and interactions with SepIVA and CwsA (Fig. S7; Table S4). Supporting this model, cells exhibiting septal NapM localization were shorter and displayed higher elongation rates than cells lacking such localization (Fig. S14B and C). The “short cells” phenotype likely reflects accelerated septation, potentially allowing cells to complete division rapidly in the presence of agents such as ethambutol, which inhibits apical growth but not cytokinesis (45). Finally, as indicated by GSEA analysis (Fig. 2D; Table S2), NapM exhibited a broad impact on vital cellular processes, including metabolism and cell envelope synthesis. Hence, its activity could inherently affect protein machineries and their functional interconnections. It remains an importnat subject for future studies to determine whether the observed effects are strictly transcriptomic or if NapM also performs a direct structural role or protein-protein interaction role within these pathways.

**Fig 7:**
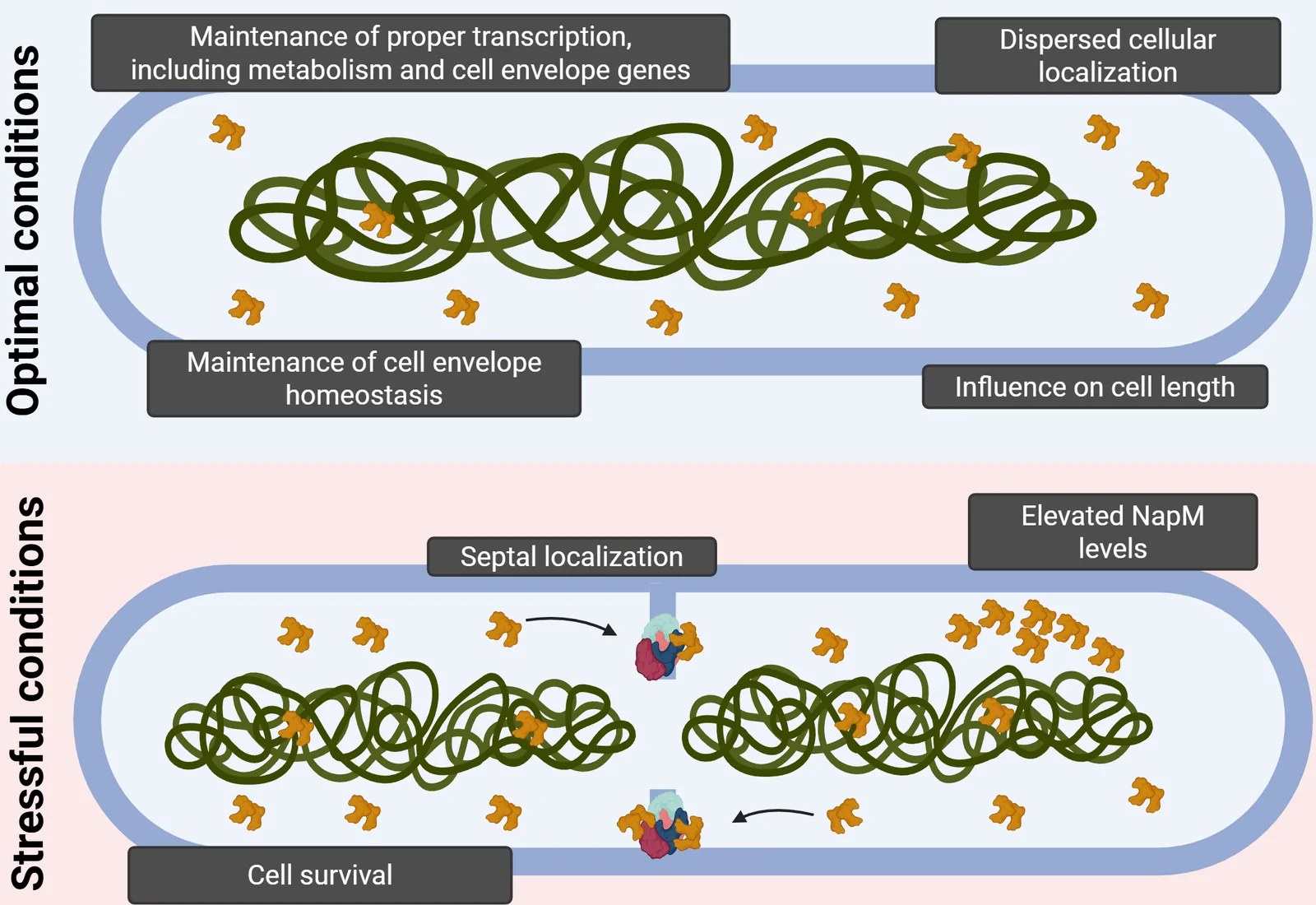
Proposed model for the dual functionality of NapM in *M. smegmatis*. Under optimal conditions, NapM modulates the transcriptomic profile to maintain cell envelope homeostasis and regulate cell length. Under stress conditions, NapM is required for cell survival; its levels increase, and the protein relocalizes to the septum, where it associates with divisome components.

Together, these observations indicate that NapM does not exhibit the defining characteristics of a canonical NAP. Rather than functioning primarily as a chromosomal architectural factor, NapM appears to influence multiple processes related to cell envelope biogenesis and cell division, although its precise mechanistic role remains to be fully elucidated (Fig. 7).

## MATERIALS AND METHODS

### RNA isolation

Total RNA was isolated as previously described (46). Cells were cultured in 50 mL to an OD_600_ = 0.8–1.2, pelleted, resuspended in 300 μL DEPC H_2_O, transferred to a clean Eppendorf tube, and mixed with 900 μL TRIzol LS (Invitrogen) at a 1:3 ratio. After being briefly vortexed, the mixture was transferred to a new tube containing zirconia beads compatible with the MP FastPrep24 homogenizer (VWR). The sample was cooled on ice for 5 minutes and then homogenized (2 × 45 s; 6 m/s) with intermittent cooling on ice. The lysate was cleared by centrifugation (12,000 × *g*, 5–7 minutes, 4°C), and the supernatant was transferred to a new Eppendorf tube. To obtain the water-soluble fraction, the supernatant was incubated for 5 minutes at room temperature in a laminar flow hood. Chloroform (250 μL) was added, and the mixture was vortexed, placed on ice, mixed vigorously for ~2 minutes, incubated on ice for 10 minutes, and centrifuged (12,000 × *g*, 4°C, 15 min). The aqueous phase (upper layer) was transferred to a new Eppendorf tube and supplemented with isopropanol (1V), sodium acetate (1:10, 5M, pH 5.2), and Glyco Blue co-precipitant (Thermo Fisher Scientific), the mixture was vortexed, and RNA was precipitated for 2 days at −80°C. The tube was centrifuged (20,000 × *g*, 4°C, 30 min), and the precipitated RNA was resuspended with 500 μL of 70% ethanol (prepared with DEPC-treated water) and pelleted by centrifugation (20,000 × *g*, 4°C, 30 min). The obtained RNA pellet was air-dried in an open Eppendorf tube, suspended in 50–55 μL DEPC-treated water, and either stored at −80°C or immediately subjected to DNase digestion. DNase digestion was performed using 10 μg of RNA in a total volume of 50 μL at 37°C for 15 minutes with DNase I (A&A Biot). The RNA was then purified using magnetic beads (MagBind), eluted with 30 µL DEPC-treated water supplemented with RiboLock RNase inhibitor (Thermo Fisher Scientific), and stored at −80°C for further procedures.

### RNA-seq

Total RNA of each analyzed strain was subjected to rRNA depletion utilizing the Pan-Actinobacteria riboPOOL (siTOOLs BIOTECH) and its corresponding protocol. Subsequently, the KAPA RNA HyperPrep kit (KK8544, ROCHE) was employed following the manufacturer’s instructions. Illumina system-compatible adapters, incorporating sample-specific 8-nucleotide-long barcoding sequences, were ligated, and cDNA libraries were PCR amplified. The resulting sequencing libraries were assessed on an Agilent 2100 Bioanalyzer with a DNA 1000 chip and quantified through real-time PCR using the NEBNext Library Quant kit for Illumina (New England Biolabs). Raw sequencing data were generated on a NextSeq500 platform (Illumina) using paired-end 75-cycle sequencing run-compatible reagents (150 cycles, NextSeq 500/550 Mid Output v2 sequencing kit; Illumina).

For analysis of RNA-seq data, the initial processing involved demultiplexing and removal of library adapters (implemented with Cutadapt v.1.3) (47). A filtering step was used to exclude short reads (<20 bp) and those with low quality (<30%) using the Sickle script v.1.33 (48). The subsequent alignment of high-quality reads to the *M. smegmatis* mc^2^ 155 genome (retrieved from NCBI, accession number NC_008596) was performed using the Bowtie2 short-read aligner (49). The resulting BAM files, containing read-mapping coordinates, were indexed and sorted with SAMtools v.1.7 (50) for data visualization via Integrative Genomics Viewer (49) and mapping of read counts to gene features with HTSeq.count script (51). The read counts from individual samples were consolidated into a count matrix. Initial filtering was applied to retain only genes with at least 10 raw counts in at least two samples. Differential gene expression analysis was performed using the DESeq2 R package with default settings and a Wald test (52). Genes were considered differentially expressed (DEGs) if they had an adjusted *P*-value (*P*adj) of ≤0.05 and an absolute log2 fold change of ≥1.585. For functional analysis, a Gene Set Enrichment Analysis (GSEA) was conducted to identify affected biological pathways. The analysis was performed using the gseKEGG function from the clusterProfiler R package against the *M. smegmatis* (msm) KEGG database and KEGG orthology (ko) in case of missing pathways in msm, with the Wald statistic serving as the ranking metric (53). Pathways were considered significantly enriched with a *P* adjusted value (*q*-value) of ≤0.25 (32). PCA was performed on Variance-Stabilizing Transformed (VST) counts for quality control and sample exploration (54). All analyses were performed in R version 4.3.1 using DESeq2 (v1.40.2) and clusterProfiler (v4.8.1). The volcano plot, visualizing the results of the DEG analysis, was created using the ggplot2 R package with the ggrepel extension (55). The chromosomal distribution of DEGs was visualized using a Circular Genome Plot created with the ggplot2 and RColorBrewer packages. The visualization of GSEA results was presented using Ridgeplots.

Raw sequence reads and processed data (count matrix) were deposited in the Gene Expression Omnibus (GEO) repository under accession number GSE275852.

### Fluorescence microscopy

To visualize NapM fluorescence fusion proteins, snapshot analysis was performed on exponential-phase *M. smegmatis* cells (OD_600_ = 0.8–1.2). Overnight cultures of *M. smegmatis* were grown in 7H9 medium supplemented with ADC and 0.05% Tween 80. The culture was centrifuged at 5,000 × *g* for 5 minutes at room temperature, and the pellet was washed once with PBS and resuspended in PBS. For cell membrane visualization, log-phase cells (OD_600_ = 0.8–1.2) were stained with FM5-95 dye (0.5 µg/mL; Thermo Fisher Scientific). For cell wall visualization, 6-TMR Tre (100 µM; Torcis Bio-Techne) was used, and for chromosomal staining, Hoechst 33342 (1 µg/mL; VWR) was applied. In each case, 1 mL of cells was incubated with the dye for 30 minutes at 37°C with continuous agitation at 180 rpm, followed by centrifugation at 5,000 × *g* for 5 minutes at room temperature. The pellet was then washed with PBS, and the cells were resuspended in PBS. All samples were mounted on Teflon-coated glass slides with 1.2% agarose and examined using a Leica DM6 epifluorescence microscope equipped with a HC PL FLUOTAR 100×/1.32 OIL PH3 objective. The following imaging parameters were applied: FM5-95 and 6-TMR Tre (excitation filter 570–590 nm, emission filter 602–662 nm) exposure time 300 ms, excitation light intensity 40%; Hoechst 33342 (excitation filter 325–375 nm, emission filter 435–485 nm) exposure time 80 ms, excitation light intensity 32%; NapM mNG (excitation filter 450–490 nm, emission filter 500–550 nm) exposure time 100 ms, excitation light intensity 32%. Images were analyzed using Fiji software (56) and R software (R Foundation for Statistical Computing, Austria; http://www.r-project.org) with the ggplot2 package.

### Time-lapse fluorescence microscopy

Overnight liquid cultures of *Mycobacterium smegmatis* (OD_600_ ~ 0.5) were observed using an ONIX microfluidics system. Briefly, 70 µL of bacterial culture was introduced into the cell inlet well of the ONIX B04A plate (Merck). For each experiment, the first two wells were flushed with PBS, and 150–300 µL of 7H9 medium supplemented with ADC and 0.05% Tween80 was loaded into each well. Once the cells were loaded into the observation chamber, they were washed for 45 minutes with the medium under 3 psi. Subsequently, the cells were cultivated for 24 hours under 1.5 psi. Where required, cells were exposed to antibiotics for 6 hours under 1.5 psi, after which the antibiotic was removed by washing with the previously used medium. To induce nutrient-restricted conditions, the flow of the medium was halted after the washing step. Images were acquired using phase contrast and the appropriate filters (475/28 nm excitation and 525/48 nm emission for mNeonGreen; 575/25 nm excitation and 625/45 nm emission for mCherry) under the following conditions: for mCherry, 50–100 ms exposure time and 50% excitation light intensity; and for mNeonGreen, 80–200 ms exposure time and 32% excitation light intensity. Images were captured automatically at 10-minute intervals using a DeltaVision Elite inverted microscope equipped with a UPlanFL N 100×/1.3 Oil Ph3 objective and an environmental chamber maintaining temperature at 37°C. Time-lapse images were analyzed using Fiji software (56) and R (R Foundation for Statistical Computing, Austria; http://www.r-project.org), with data visualization performed using the ggplot2 package.

### Lattice SIM

Exponential-phase cells (OD_600_ = 0.8–1.2) exposed to ethambutol were imaged using an Elyra 7 (Zeiss) inverted microscope equipped with an sCMOS 4.2 CL HS camera and an alpha Plan-Apochromat 100×/1.46 Oil DIC M27 objective, along with an Optovar 1.6× magnification changer. Fluorescence was excited using a 488 nm laser (100 mW), and signals were captured through a multiple-beam splitter (405/488/561/641 nm) and corresponding laser-block filters (405/488/561/641 nm). Samples were prepared on agar pads (1% agarose in Milli-Q water, poured into 1.0 × 1.0 cm GeneFrames; Thermo Fisher Scientific). Cells were illuminated with a 488 nm laser at 5% intensity and a 30 ms exposure time in Lattice-SIM mode, consisting of 15 phases. Image reconstruction was performed using ZEN 3.0 SR software (Zeiss) with standard parameters. Images were analyzed using Fiji software (56).

### Bacterial two-hybrid system

To verify the dimerization of *Mycobacterium smegmatis* NapM, we employed the Bacterial Adenylate Cyclase Two-Hybrid System (BACTH System Kit, EUROMEDEX). Briefly, derivatives of pUT18, pUT18C, pKT25, and pKNT25 were generated through PCR amplification using primers BTH_xbaI_napM_Fw and BTH_kpnI_napM_Rv (Table S5). The resulting products were initially cloned into the pGEM-T Easy vector (Promega) and then subcloned into backbone vectors. The experimental procedures were conducted according to the manufacturer’s guidelines provided in the BACTH System Kit. Transformants were plated on LB agar plates supplemented with IPTG, kanamycin, ampicillin, and X-gal and incubated for 2 days at 30°C. Plate images were captured using a custom-made platform for the visualization of plates.

### Total lipid extraction and TLC analysis

Total lipids were extracted from dry (50 mg) and wet (400 mg) cell masses of *Mycobacterium smegmatis* WT and Δ*napM* strains. Each collected cell pellet was dissolved in 1 mL of MQ water and subjected to chloroform-methanol (1:2, vol/vol) extraction. The combined extracts were partitioned in a chloroform-methanol-water mixture (1:2:0.8, vol/vol/vol), and the organic phase was collected and dried at 40°C under a stream of nitrogen.

The obtained total lipids were dissolved in chloroform to a final concentration of 50 mg/mL. Equal amounts of lipid extracts were applied to silica gel 60-precoated HPTLC or TLC plates (Merck; layer thickness, 0.2 mm). Lipids were analyzed using TLC with a chloroform-methanol-water (65:25:4, vol/vol/vol) solvent system. For lipid visualization, TLC plates were revealed with 0.5% vanillin in ethanol with 3% H_2_SO_4_, followed by heating at 120°C.

### MALDI-TOF MS

MALDI-TOF mass spectrometry was performed using an ultrafleXtreme spectrometer (Bruker). Spectra were measured in reflectron negative ion mode with the following parameters: ion source 1, 20.0 kV; ion source 2, 17.95 kV; lens, 6.5 kV; reflector, 21.10 kV; and reflector 2, 10.72 kV. The mass range analyzed was 700–3,500 Da, using flexControl 3.4 software (Bruker). Approximately 2,000 laser shots were totaled from one spot. The flexAnalysis 3.4 software (Bruker) was used for data analysis. Norharman (β-carboline) was used as the matrix.

### Statistical analysis and data visualization

Each experiment (except RNA-seq) was conducted with three biological replicates for each strain. Levene’s test was performed in R Studio (R Foundation for Statistical Computing, Austria; http://www.r-project.org) using the *car* package to assess variance equality, and depending on the results, either Student’s *t*-test (for equal variances) or Welch’s *t*-test (for unequal variances) was applied. Data sets not subjected to statistical testing and RNA-seq data were not analyzed with Levene’s test. All reported ± values represent the standard deviation (SD) calculated from the indicated number of biological replicates, and all presented plots include the SD error bars. Statistical analyses and visualizations were carried out in RStudio using the *ggplot2* and *stats* packages (R Foundation for Statistical Computing, Austria; http://www.r-project.org). Significance levels were defined as follows: n.s (not significant), *P < 0.05, **P < 0.01, ***P < 0.001, and ****P < 0.0001. Additional data visualizations were created using BioRender (https://www.biorender.com/) under an Academic License.

## Data Availability

The original contributions presented in the study are included in the article and supplemental material. RNA-seq data were deposited in the Gene Expression Omnibus (GEO), a public repository for functional genomics data, and are available under accession number GSE275852. The mass spectrometry proteomics data were deposited in the ProteomeXchange Consortium via the PRIDE partner repository with the data set identifier PXD062473. Further inquiries can be directed to the corresponding authors. The AlphaFold3-predicted structural models of the NapM protein and its complexes with dsDNA and DivIVA from *M. smegmatis* have been deposited in Figshare under the DOI: 10.6084/m9.figshare.30563126.

## SUPPLEMENTAL MATERIAL

Supplemental figures spectrum.03938-25-s0001.pdf Fig. S1 to S15.

Table S1 spectrum.03938-25-s0002.txt Differentially expressed genes in ∆napM strain.

Table S2 spectrum.03938-25-s0003.txt Enriched pathways in ∆napM strain.

Supplemental tables spectrum.03938-25-s0004.docx Tables S3 to S7.

Supplemental material spectrum.03938-25-s0005.docx Experimental procedures.

Movie S1 spectrum.03938-25-s0006.mp4 Time-lapse imaging of NapM-mNeonGreen cells using ONIX microfluidics system during EMB exposure without antibiotic washout. Arrows indicate septal localization of NapM-mNeonGreen. Images were acquired automatically every 10 min. Scale bar: 5 μm. Time-lapse images were processed in ImageJ using LUT: Smart.

Movie S2 spectrum.03938-25-s0007.mp4 Time-lapse imaging of NapM-mNeonGreen↑ cells using ONIX microfluidics system upon 6 h EMB exposure, followed by antibiotic washing. Yellow arrows indicate septum-like localization of NapM-mNeonGreen. Images were acquired automatically every 10 min. Scale bar: 1 μm.

Movie S3 spectrum.03938-25-s0008.mp4 Time-lapse imaging of NapM-mNeonGreen cells producing additional copy of DivIVA fused with mCherry using ONIX microfluidics system upon 8 hours EMB exposure, followed by antibiotic washing. Arrows indicate septal localization of NapM-mNeonGreen (green arrows) or DivIVA-mCherry (red arrows). Images were acquired automatically every 10 min. Scale bar: 1 μm.

ASM does not own the copyrights to Supplemental Material that may be linked to, or accessed through, an article. The authors have granted ASM a non-exclusive, world-wide license to publish the Supplemental Material files. Please contact the corresponding author directly for reuse.
