# Supplementary material for: The mycobacterial nucleoid-associated protein NapM exhibits stress-induced septal localization and modulates cell envelope gene expression
Source: Microbiol Spectr. 2026 Jun 9;14(7):e03938-25. doi: 10.1128/spectrum.03938-25 (PMC13340245; doi:10.1128/spectrum.03938-25)
Supplement: Supplemental figures — Fig. S1 to S15. [file spectrum.03938-25-s0001.pdf]

A

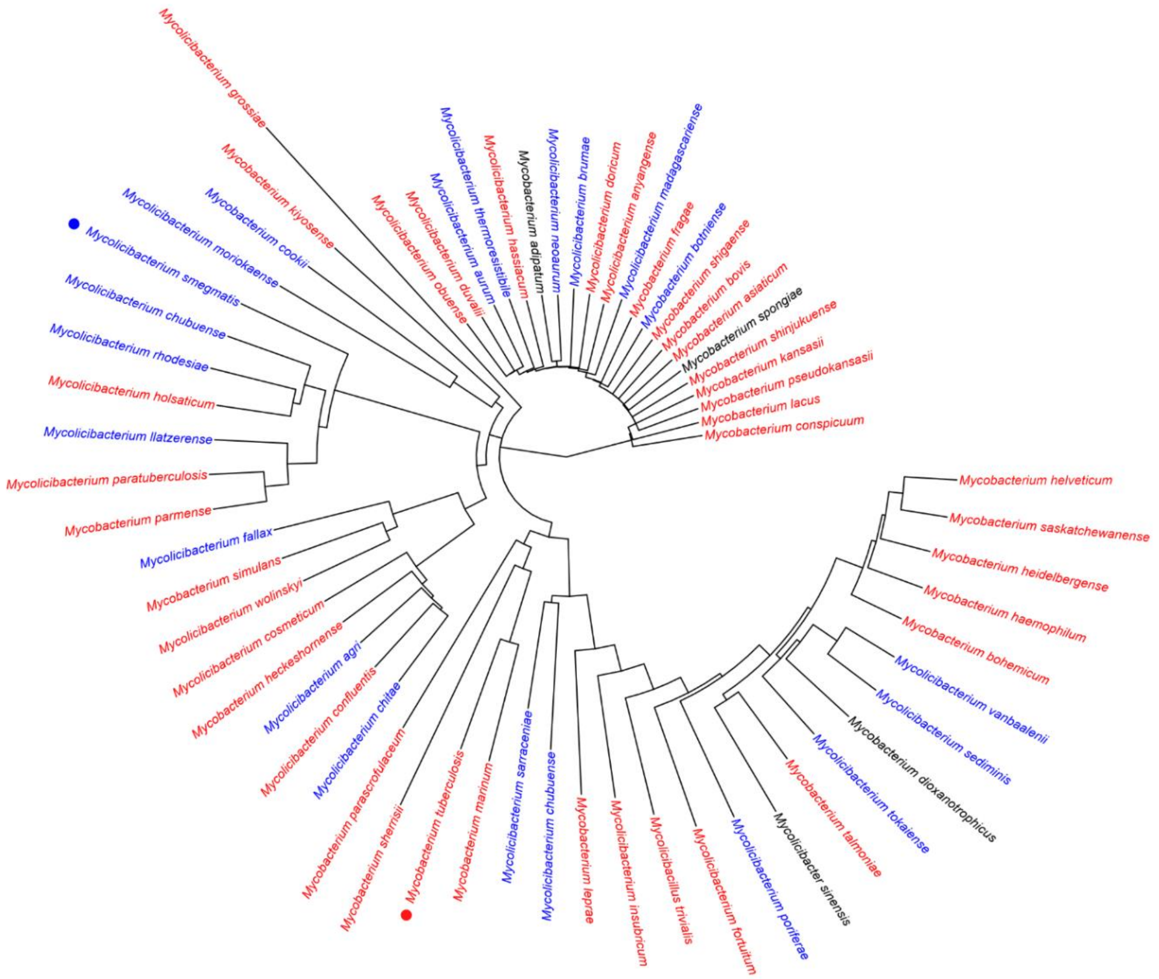
Mycolicibacterium grossiae
Mycobacterium kiyosense
Mycobacterium cookii
Mycolicibacterium moriokaense
Mycolicibacterium smegmatis
Mycolicibacterium chubuense
Mycolicibacterium rhodesiae
Mycolicibacterium holsaticum
Mycolicibacterium llatzerense
Mycolicibacterium paratuberculosis
Mycobacterium parmense
Mycolicibacterium fallax
Mycobacterium simulans
Mycolicibacterium wolinskyi
Mycolicibacterium cosmeticum
Mycobacterium heckeshornense
Mycolicibacterium agri
Mycolicibacterium confluentis
Mycolicibacterium chitae
Mycobacterium parascrofulaceum
Mycobacterium sherrisii
Mycobacterium tuberculosis
Mycobacterium marinum
Mycolicibacterium sarraceniae
Mycolicibacterium chubuense
Mycobacterium leprae
Mycolicibacterium insubricum
Mycolicibacillus trivialis
Mycolicibacterium fortuitum
Mycolicibacterium poriferae
Mycolicibacter sinensis
Mycobacterium talmoniae
Mycolicibacterium tokaiense
Mycobacterium dioxanotrophicus
Mycolicibacterium sediminis
Mycolicibacterium vanbaalenii
Mycobacterium bohemicum
Mycobacterium haemophilum
Mycobacterium heidelbergense
Mycobacterium saskatchewanense
Mycobacterium helveticum
Mycobacterium conspicuum
Mycobacterium lacus
Mycobacterium pseudokansasii
Mycobacterium kansasii
Mycobacterium shinjukuense
Mycobacterium spongiae
Mycobacterium asiaticum
Mycobacterium bovis
Mycobacterium shigaense
Mycobacterium botniense
Mycobacterium fragae
Mycolicibacterium madagascariense
Mycobacterium anyangense
Mycolicibacterium doricum
Mycolicibacterium brumae
Mycolicibacterium neoaurum
Mycobacterium adipatum
Mycolicibacterium hassiacum
Mycolicibacterium thermoresistibile
Mycolicibacterium aurum
Mycolicibacterium duvalii
Mycolicibacterium obuense

B

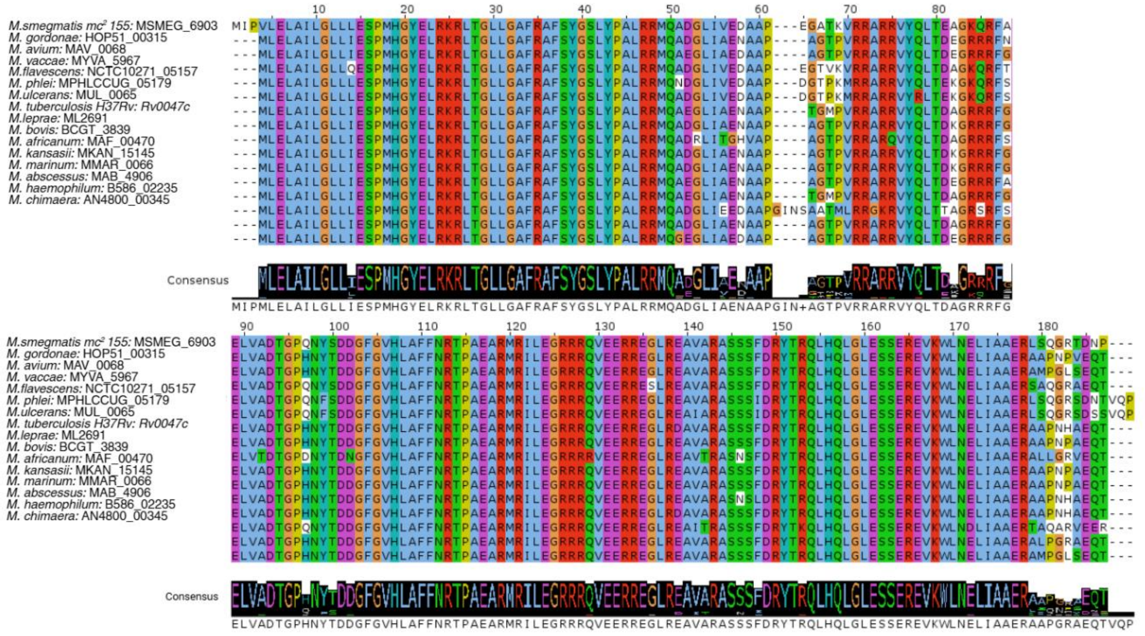
M.smegmatis mc² 155: MSMEG_6903
M. gordonae: HOP51_00315
M. avium: MAV_0068
M. vaccae: MYVA_5967
M.flavescens: NCTC10271_05157
M. phlei: MPHLCCUG_05179
M.ulcerans: MUL_0065
M. tuberculosis H37Rv: Rv0047c
M.leprae: ML2691
M. bovis: BCGT_3839
M. africanum: MAF_00470
M. kansasii: MKAN_15145
M. marinum: MMAR_0066
M. abscessus: MAB_4906
M. haemophilum: B586_02235
M. chimaera: AN4800_00345
Consensus
MIPMLELAILGLLIIESPMHGYELRKRLTGLLGAFRAFSYGSLYPALRRMQADGLIAENAAPGIN+AGTPVRRARRVYQLTDAGRRRFG
ELVADTGPHNYTDDGFGVHLAFFNRTPAEARMRILEGRRRQVEERREGLREAVARASSSFDRYTRQLHQLGLESSEREVKWLNELIAAERAAPGRAEQTVQP

Figure S1. Phylogenetic relationships and sequence diversity of NapM homologs. (A) Neighbor-joining phylogenetic tree of NapM homologs from the family *Mycobacteriaceae*, based on MAFFT alignment. Tip labels are italicized and color-coded by lifestyle / source of isolate (red = pathogen, blue = saprophyte, grey = unknown). Red dot point to *M. tuberculosis* while blue dot to *M. smegmatis*. **(B)** Multiple sequence alignment of representative NapM sequences illustrating conserved regions and diversity at the C-terminus; consensus sequence shown below.

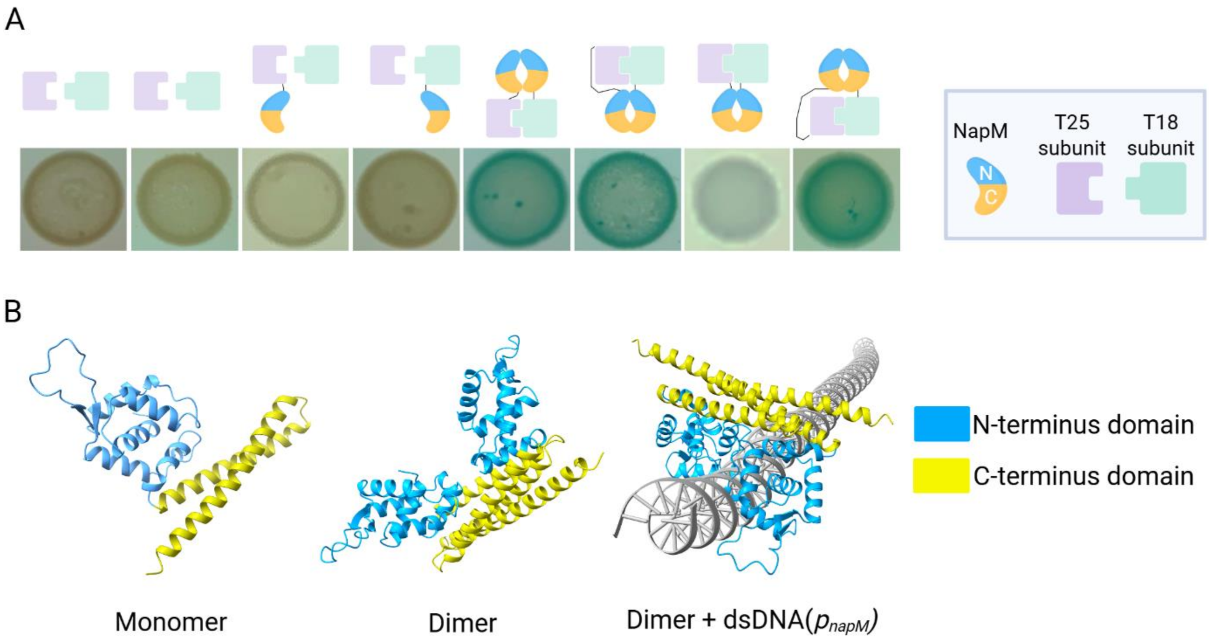

Figure S2. Homodimerization of NapM. (A) Bacterial two-hybrid (BTH) assay testing interactions between *M. smegmatis* NapM fusion constructs. In all tested orientations, NapM–NapM interactions were detected, indicating self-association. Cartoon above each panel illustrates domain–fusion arrangement (T25 (purple) or T18 (green) subunits). **(B)** AlphaFold structural predictions of NapM. Shown are the monomer (left), homodimer (middle), and dimer bound to double-stranded DNA corresponding to the *napM's* promoter (right). The N-terminal domain is shown in blue and the C-terminal domain in yellow.

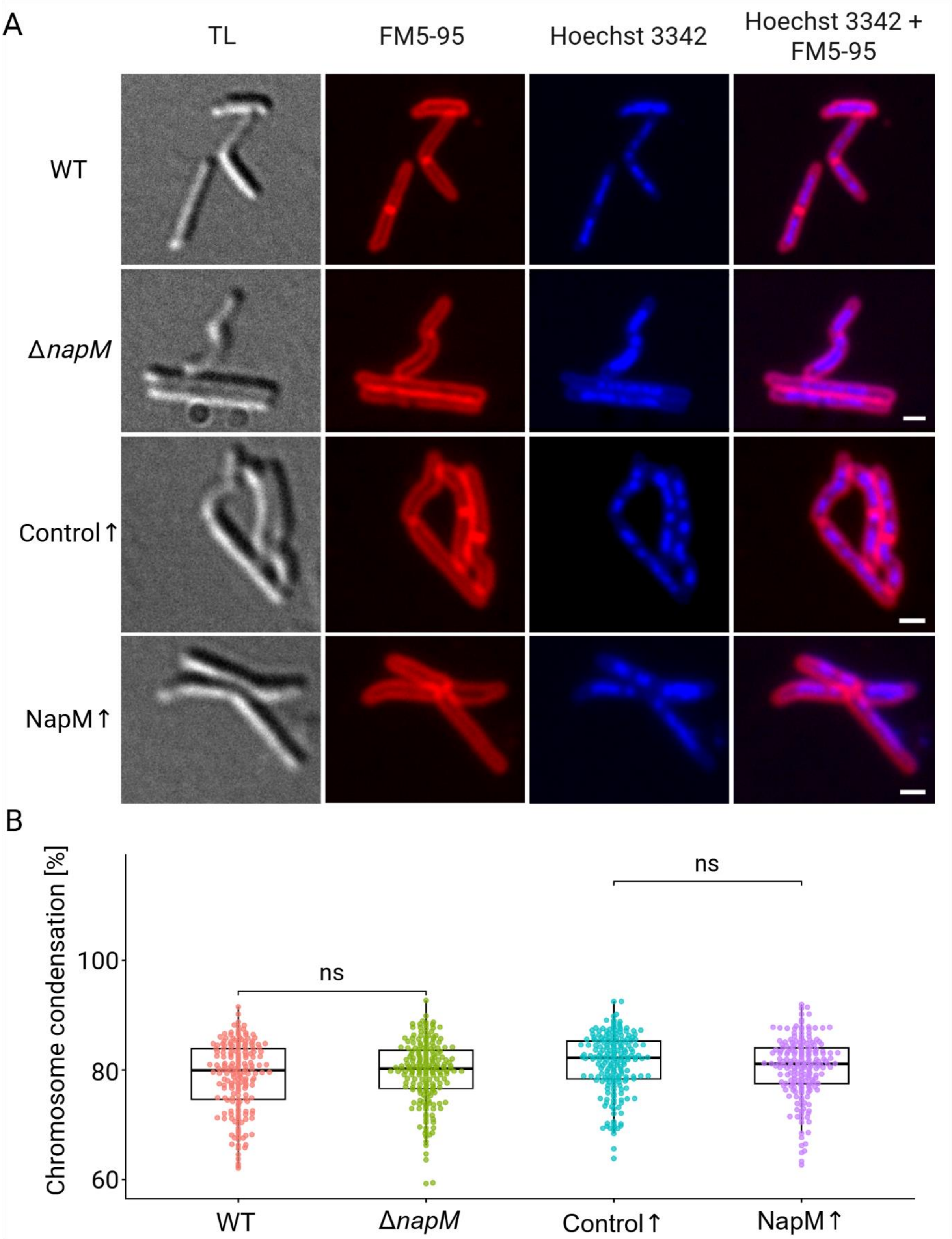

Figure S3. Phenotypic analysis of the wild-type (WT) and *napM* mutant strains. A. Nucleoid (Hoechst 3342) staining of the analyzed *M. smegmatis* strains. TL – transmitted light; FM5-95 – membrane staining. No morphological changes are observed. Scale bar, 1 µm. **B.** Boxplots presenting chromosome condensation calculated as the ratio of chromosome length

to the cell length. No differences were observed for Δ*napM* compared to WT(79.6 ± 5.8 and 78.8 ± 6.4, respectively; t(377) = -1.33, p = 0.19; n = 185 and 194, respectively), and similarly for NapM↑ compared to Control↑ (80.4 ± 5.5 and 81.2 ± 5.4, respectively; t(384) = -1.44, p = 0.15; n = 193 and 193, respectively).

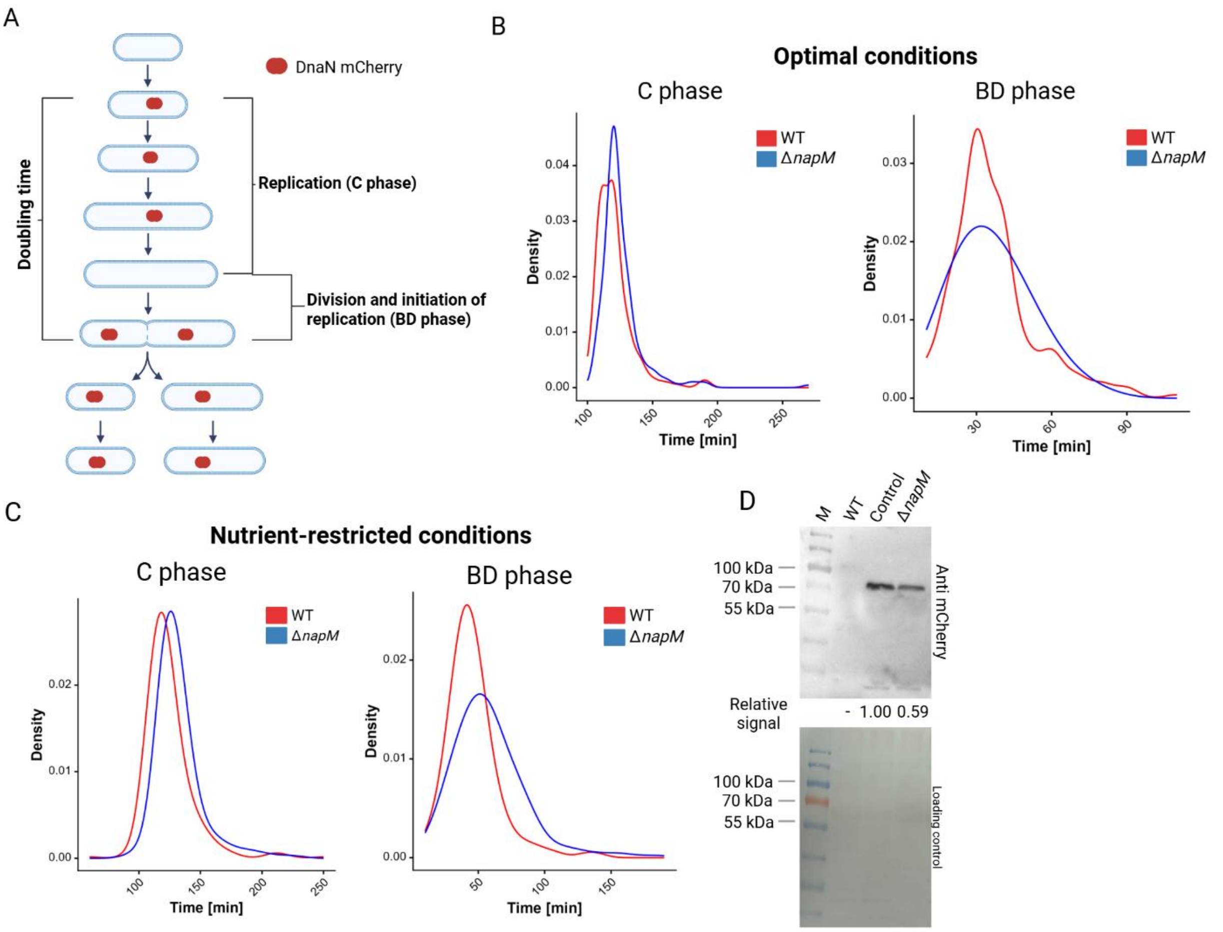

Figure S4. Analysis of replication dynamics in Δ*napM* and wild-type (WT) strains. A. Schematic representation of experimental workflow utilizing DnaN-mCherry as a replisome marker. B. Density plots presenting the duration of replication phases in ΔnapM and the wild-type (WT) strains in optimal conditions. Deletion of the napM gene results in a slight elongation of the C phase (replication) in M. smegmatis (126 ± 21 min and 120 ± 17 min, respectively; t(608) = 3.46, p = 5.40*10-4, n = 305). The second panel shows the time between termination of replication and initiation of replication in daughter cells (BD phase) is not affected by deletion of napM in compared to WT (35 ± 16 min and 36 ± 16, respectively; t(1314) = -1.50 p = 1.30*10-1, n = 658). C. Density plots presenting the duration of replication phases in ΔnapM and WT strains under nutrient-limited conditions. Deletion of napM results in an extended C phase (124 ± 19 for WT and 132 ± 18 for mutant; t(537) = 3.46, p = 3.60*10-6, n = 267 and 272) and also BD phase (46 ± 20 for WT and 58 ± 27 for mutant; t(1188) = 9.31, p < 2.22 x 10-

16, n = 628 and 652). D. Western blot analysis of DnaN levels in the DnaN-mCherry strain (Control; dnaN–mcherry gene expressed from the native locus) and the ΔnapM strain (DnaN-mCherry strain lacking the napM gene in compared with WT strain. Expected product size: ~68 kDa. Relative signal intensities were calculated by normalizing each band to the Control signal (set to 1.00).

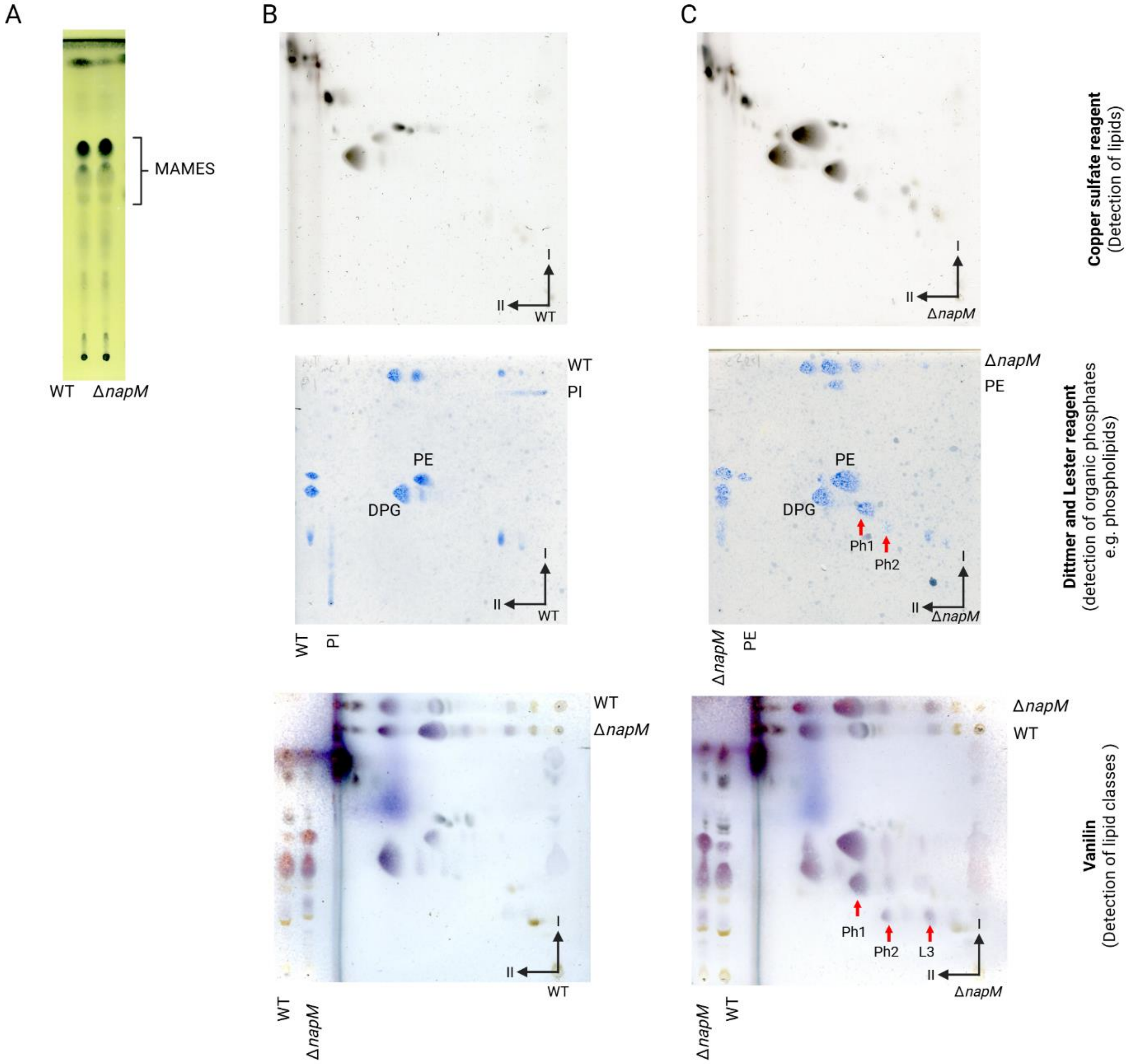

Figure S5. Thin layer chromatography (TLC) profiles of methyl esters of mycolic acids and polar lipids of the wild-type and Δ*napM* strains. A. MAMES - mycolic acids methyl esters. Solvent system: hexane-diethyl ether (85:15, v/v), three runs. Detection: 10% (w/v) molybdophosphoric acid in ethanol. Two-dimensional TLC (2D-TLC) analysis of lipids extracted from WT (**B**) and *ΔnapM* strain (**C**). **Solvent systems:** I – chloroform–methanol–water (65:25:4, v/v/v); II – chloroform–acetic acid–methanol–water (80:15:12:4, v/v/v/v).

Detection: copper sulfate reagent (lipids), Dittmsser & Lester (phospholipids), vanillin (lipid classes). Red arrows indicate additional phospholipid spots (Ph1, Ph2) and a lipid species (L3) detected in the *ΔnapM* strain. DPG – diphosphatidylglycerol, PE - phosphatidylethanolamine

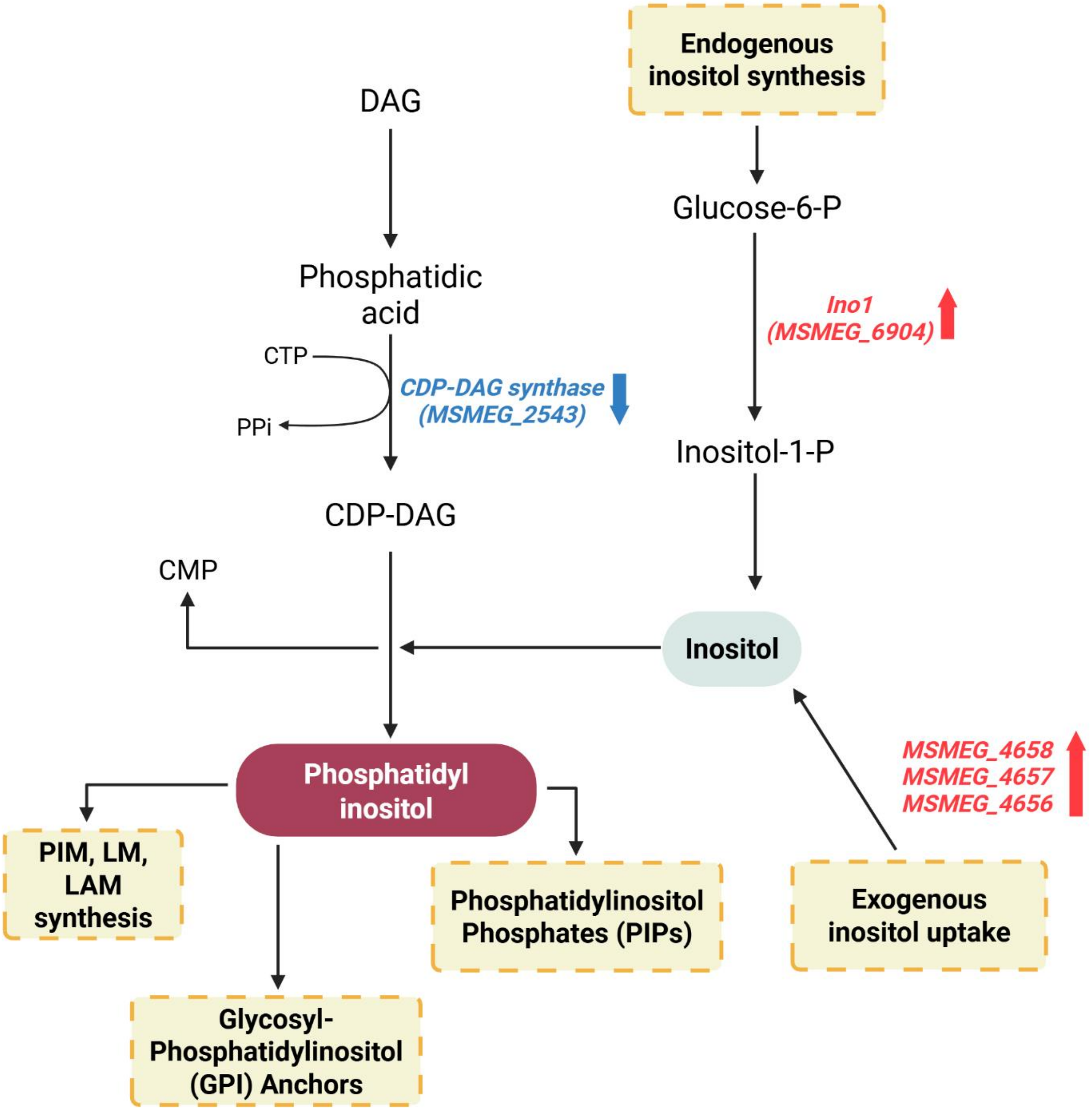

Figure S6. Transcriptomic changes in the inositol and phosphatidylinositol biosynthesis pathways in *M. smegmatis ΔnapM* strain based on DEG and GSEA analysis. Blue indicates downregulation in the Δ*napM* strain, while red represents upregulation.

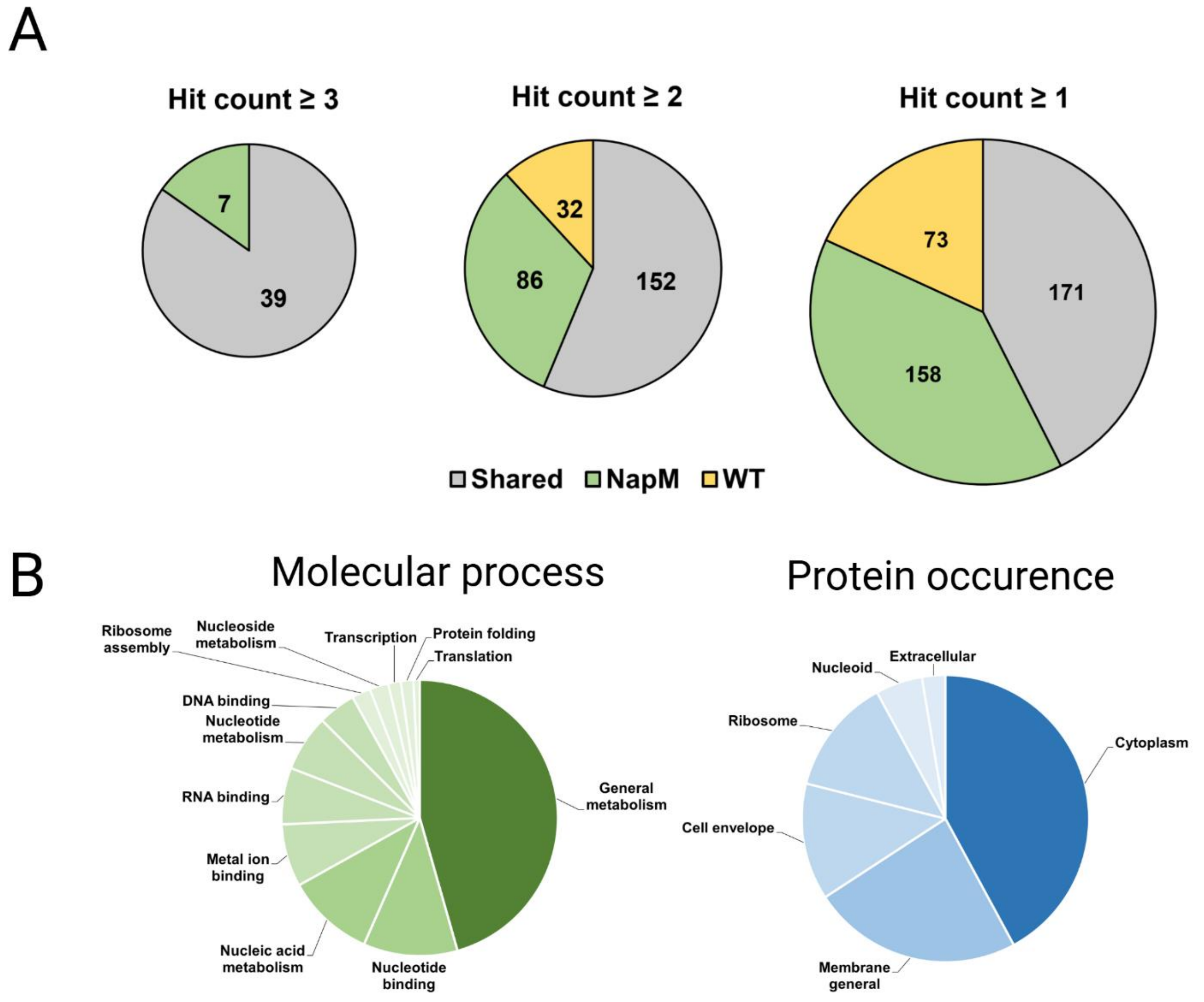

Figure S7. Comparative proteomic analysis of NapM-FLAG and WT strains of *M. smegmatis*. A. Venn pie charts showing the number of unique and shared proteins identified in NapM-FLAG (green), wild-type (WT; yellow), and shared (gray) proteomes at different detection stringency thresholds: proteins detected in ≥3 replicates (left), ≥2 replicates (middle), and ≥1 replicate (right). **B**. Functional classification of proteins uniquely identified in the NapM mutant. Left: Categorization based on molecular processes, with the majority of proteins involved in general metabolism, nucleotide binding, and nucleic acid interactions. Right: Subcellular localization of these proteins, showing a predominance of cytoplasmic proteins, followed by membrane-associated and ribosomal proteins.

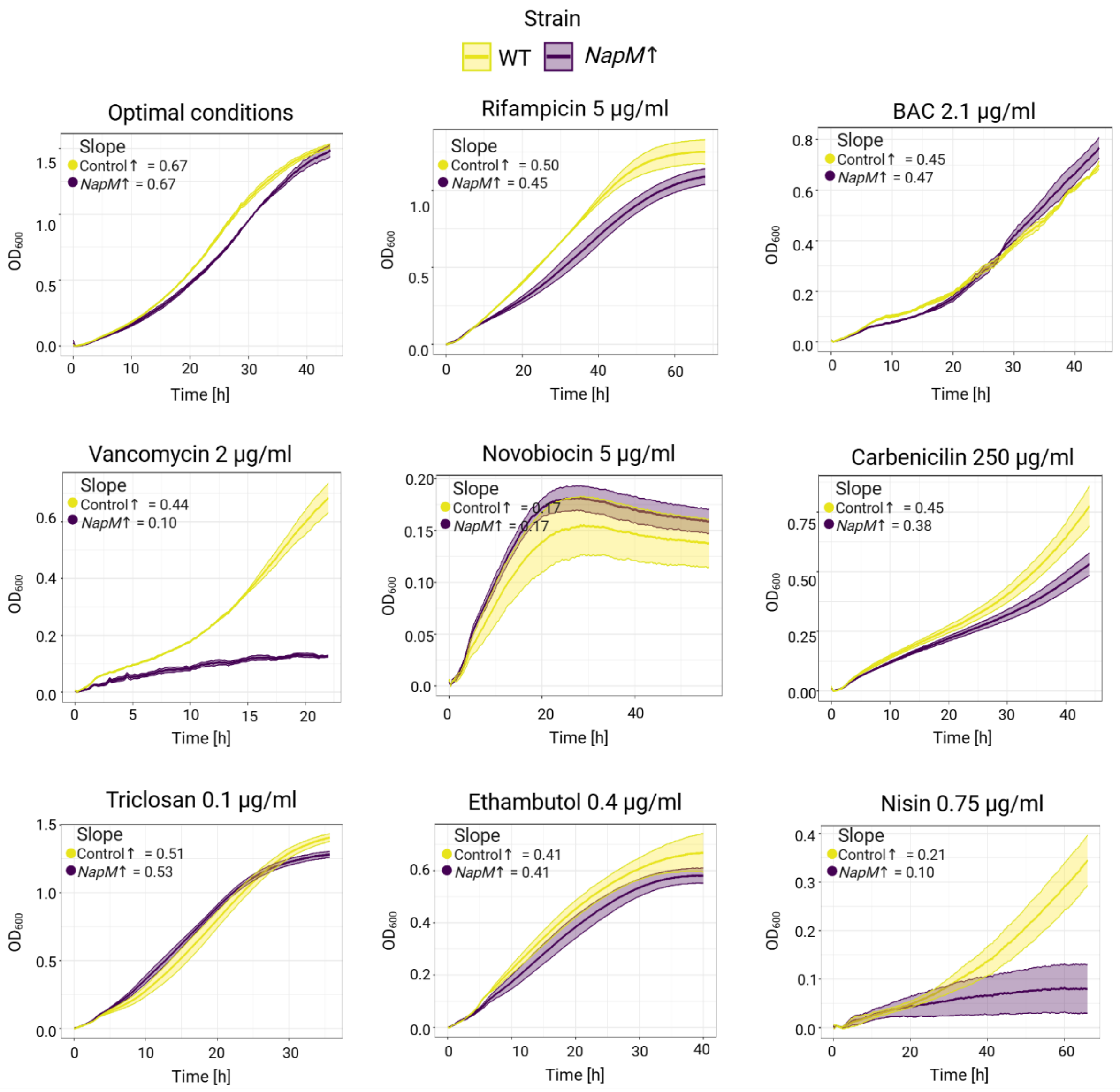

Figure S8. Analysis of growth rates in *M. smegmatis* cells overproducing NapM under various stress conditions. Growth curves of the NapM↑ strain compared to the Control↑ strain.

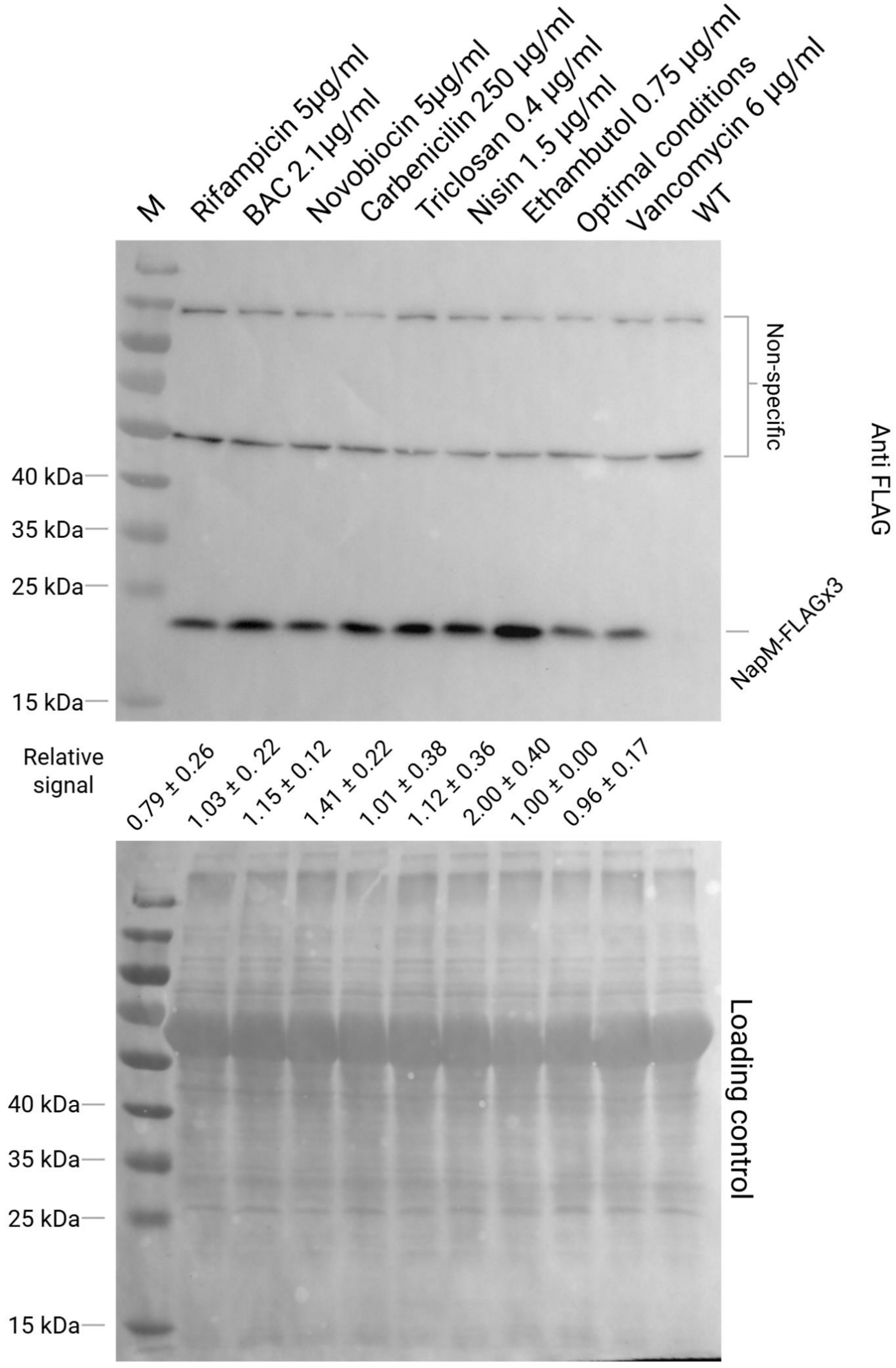
M
Rifampicin 5μg/ml
BAC 2.1μg/ml
Novobiocin 5μg/ml
Carbenicilin 250 μg/ml
Triclosan 0.4 μg/ml
Nisin 1.5 μg/ml
Ethambutol 0.75 μg/ml
Optimal conditions
Vancomycin 6 μg/ml
WT
Non-specific
Anti FLAG
40 kDa
35 kDa
25 kDa
15 kDa
NapM-FLAGx3
Relative signal
0.79 ± 0.26
1.03 ± 0. 22
1.15 ± 0.12
1.41 ± 0.22
1.01 ± 0.38
1.12 ± 0.36
2.00 ± 0.40
1.00 ± 0.00
0.96 ± 0.17
40 kDa
35 kDa
25 kDa
15 kDa
Loading control

Figure S9. Western blot analysis of NapM-FLAGx3 protein which tend to accumulate in *M. smegmatis* cells upon stress exposition. Estimated NapM-FLAGx3 protein size is 24 kDa. Relative signal intensities with SD were calculated by normalizing each band to the signal under optimal conditions (set to 1.00). The upper bands – around 55 kDA and 130 kDA are non-specific bands as they were also detected in wild type strain.

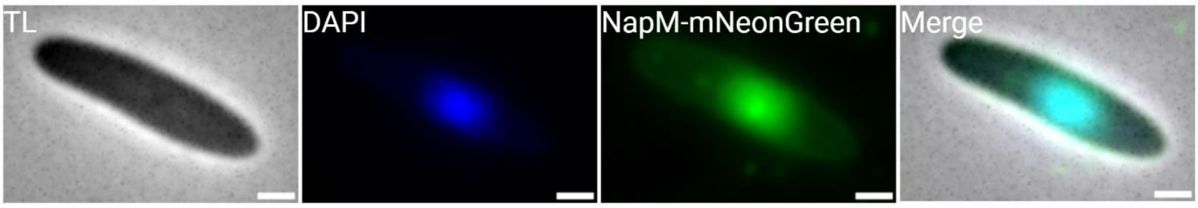

Figure S10. Colocalization of NapM-mNeonGreen with DAPI-stained nucleoid in *E. coli* cells. TL- transmitted light. Scale bar, 1 μm

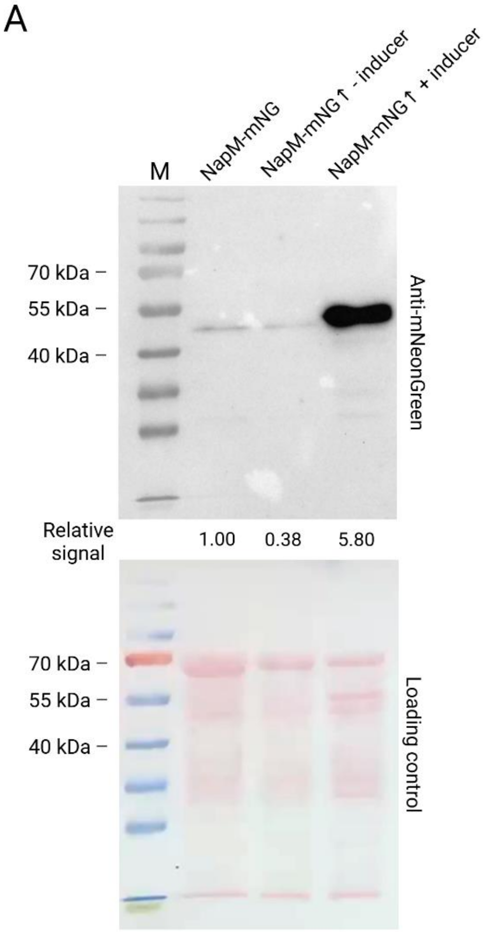

Figure S11. Western blot analysis of NapM-mNeonGreen and NapM-mNeonGreen↑ strains. Comparison of the levels of NapM-mNeonGreen fusion protein produced in strains expressing *napM-mneongreen* gene under native (NapM-mNeonGreen strain) and inducible

promoter (NapM-mNeonGreen↑ strain). Relative signal intensities were calculated by normalizing each band to the signal of NapM-mNeonGreen strain (set to 1.00). Estimated NapM-mNeonGreen protein size is 50 kDa.

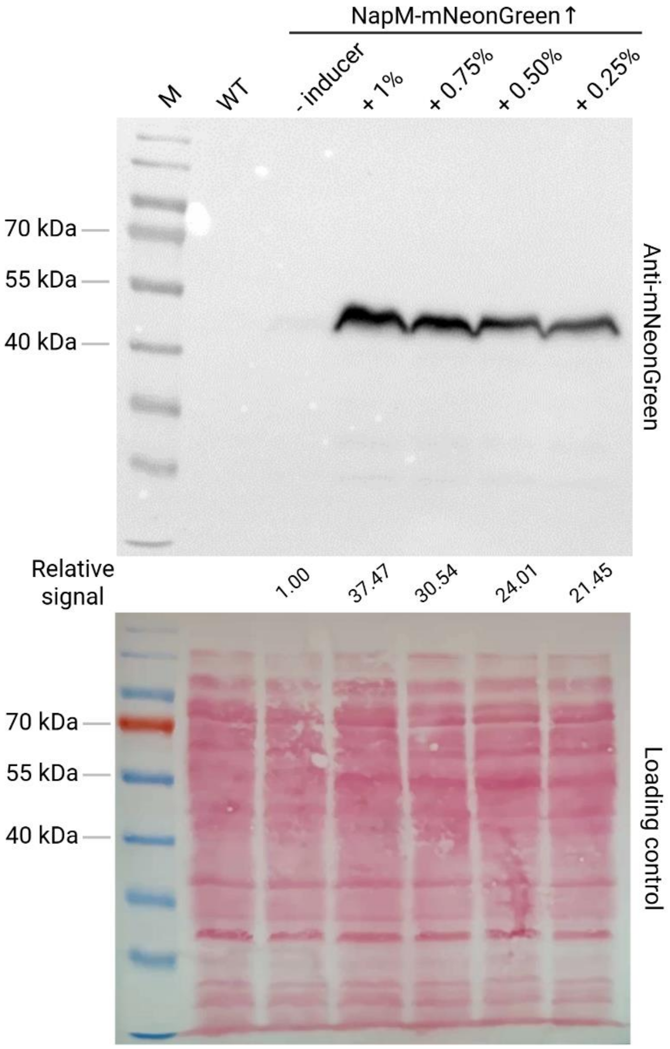

Figure S12. Western blot analysis of NapM-mNeonGreen fusion protein level in NapM-mNeonGreen↑ strain in optimal conditions with different acetamide (inducer) concentrations. Estimated NapM-mNeonGreen protein size is 50 kDa. Relative signal intensities were calculated by normalizing each band to the signal of the band in "- inducer" well (set to 1.00).

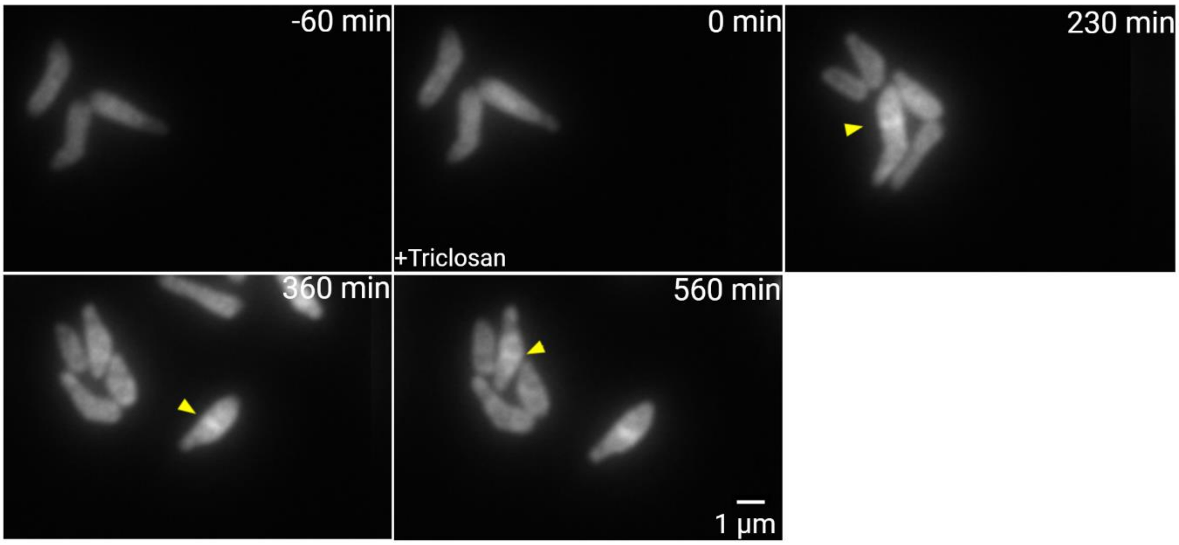

Figure S13. Time-lapse imaging of NapM-mNeonGreen↑ cells upon 6 h triclosan exposure. Yellow arrows indicate septal localization of the NapM-mNeonGreen fusion protein.

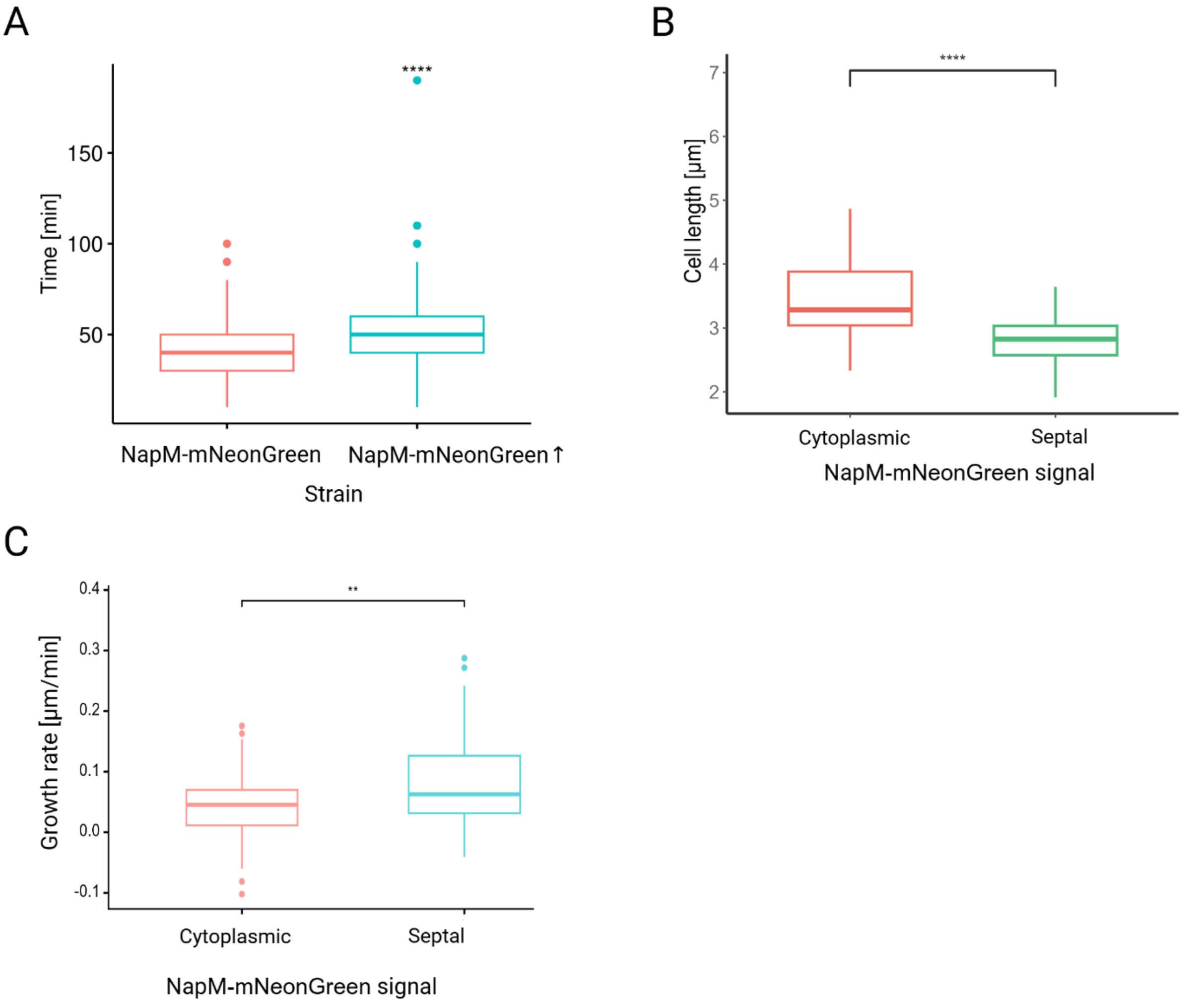

Figure S14. Analysis of NapM-mNeonGreen localization in *M. smegmatis* cells upon ethambutol (EMB) exposure. Boxplots presenting duration of NapM-mNeonGreen signal within the septum area (**A**), differences in cells length (**B**) and elongation rate (**C**) in NapM-mNeonGreen↑ strain.

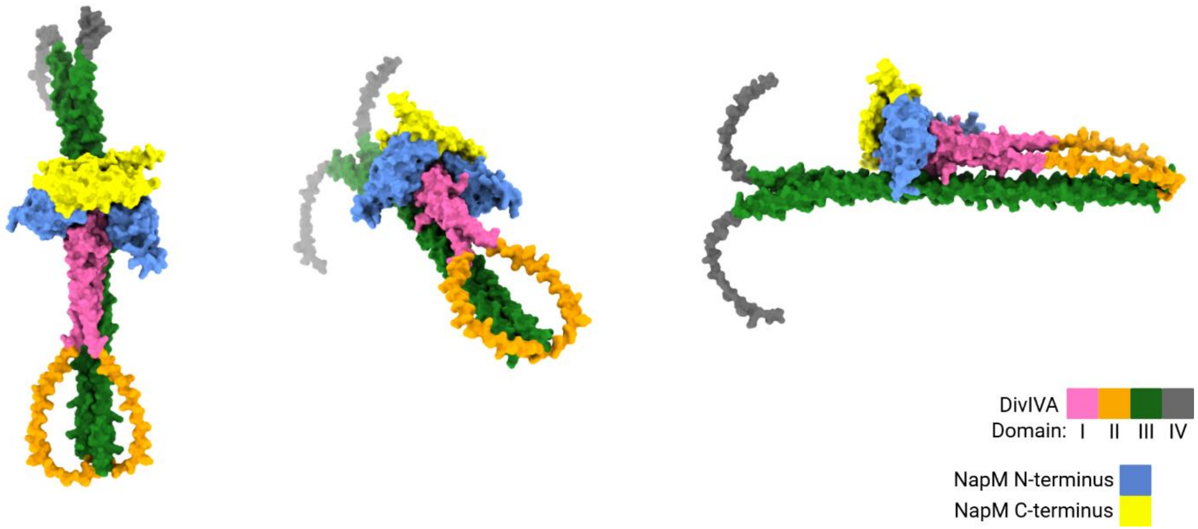

Figure S15. AlphaFold 3 prediction of tertiary structure of NapM-DivIVA oligomer. The prediction indicates a potential interaction between the NapM dimer and the N-terminal domain of the DivIVA dimer, responsible for anchoring the protein to the membrane.
