# Supplementary material for: The mycobacterial nucleoid-associated protein NapM exhibits stress-induced septal localization and modulates cell envelope gene expression
Source: Microbiol Spectr. 2026 Jun 9;14(7):e03938-25. doi: 10.1128/spectrum.03938-25 (PMC13340245; doi:10.1128/spectrum.03938-25)
Supplement: Supplemental tables — Tables S3 to S7. [file spectrum.03938-25-s0004.docx]

**Table S3.** Comparative analysis of gene expression changes for polar lipid metabolism and peptidoglycan biosynthesis in *M. smegmatis ∆napM* strain. The faint blue are genes which were not present in DEG (**Table S1**) but were part of significantly affected pathways in GSEA.

| *M. smegmatis* | *M. tuberculosis* | Function | log2FC | p "adjusted" |
| --- | --- | --- | --- | --- |
| Inositol uptake | | | | |
| MSMEG_4658 | - | inositol ABC transport system sugar-binding protein | 2.48 | 1.38*10^21^ |
| MSMEG_4657 | - | inositol ABC transport system permease protein | 3.71 | 5.85*10^32^ |
| MSMEG_4656 | - | inositol ABC transport system ATP-binding protein | 3.26 | 2.12*10^31^ |
| Inositol & PI biosynthesis | | | | |
| MSMEG_6904 | Rv0046c (ino1) | myo-inositol-1-phosphate synthase | 5.12 | 3.27*10^169^ |
| MSMEG_4190 | Rv2131c (cysQ) | inositol monophosphatase (IMP) | -1.61 | 4.40*10^10^ |
| MSMEG_2543 | Rv2881c (cdsA) | phosphatidate cytidylyltransferase (CDP-diglyceride synthetase) | -1.56 | 5.79*10^17^ |
| MSMEG_2933 | Rv2612c (pgsA) | PI synthase (phosphatidylinositol synthase) | -1.06 | 2.80*10^05^ |
| PIMs biosynthesis | | | | |
| MSMEG_2935 | Rv2610c (pimA) | alpha-mannosyltransferase | -1.34 | 1.00*10^12^ |
| MSMEG_4253 | Rv2188c (pimB) | mannosyltransferase | -1.04 | 1.85*10^5^ |
| MSMEG_2934 | Rv2611 | acyltransferase (acylation of the 6-position of the mannose of PIM1) | -1.71 | 3.01*10^16^ |
| LM and LAM precursor formation | | | | |
| MSMEG_3859 | Rv2051c (ppm1) | polyprenol-monophosphomannose synthase Ppm1 | -1.38 | 3.25*10^12^ |
| LM and LAM biosynthesis | | | | |
| MSMEG_4247 | Rv2181 (mptC) | alpha(1->2) mannosyltransferase | -1.53 | 1.72*10^15^ |
| MSMEG_0359 | Rv0236c (aftD) | arabinofuranosyltransferase | -2.07 | 3.60*10^41^ |
| Peptidoglycan biosynthesis & cell separation | | | | |
| MSMEG_0928 | Rv0482 (murB) | UDP-N-acetylmuramate dehydrogenase | -2.10 | 7.12*10^21^ |
| MSMEG_4229 | Rv2155c (murD) | UDP-N-acetylmuramoylalanin*10D-glutamate ligase | -1.22 | 1.28*10^12^ |
| MSMEG_4232 | Rv2158c (murE) | UDP-N-acetylmuramoylalanyl-D-glutamat*102.6-diaminopimelate ligase | -1.87 | 1.48*10^24^ |
| MSMEG_4231 | Rv2157c (murF) | UDP-N-acetylmuramoylalanyl-D-glutamyl-2.6-diaminopimelat*10D-alanyl-D-alanyl ligase | -1.28 | 8.05*10^16^ |
| MSMEG_4230 | Rv2156c (murX) | phospho-N-acetylmuramoyl-pentappeptidetransferase | -1.96 | 2.37*10^28^ |
| MSMEG_4227 | Rv2153c (murG) | UPD-N-acetylglucosamin*10N-acetylmuramyl-(pentapeptide) pyrophosphoryl-undecaprenol-N-acetylglucosamine transferase | -1.94 | 1.62*10^29^ |
| MSMEG_6929 | (murJ) | flippase: transbilayer movement of Lipid II during global PG synthesis | -1.21 | 1.14*10^12^ |
| MSMEG_4228 | Rv2154c (ftsW) | flippase; transbilayer movement of Lipid II during septal PG synthesis | -1.49 | 2.17*10^13^ |
| MSMEG_6900 | Rv0050 (ponA1) | transpeptidase (DD-transpeptidase); (murein polymerase) | -1.29 | 9.98*10^15^ |
| MSMEG_4233 | Rv2163 (pbpB) | penicillin-binding membrane protein; regulation of cell separation | -1.47 | 2.05*10^19^ |
| MSMEG_0651 | Rv0320 | endopeptidase; PG hydrolase | -1.21 | 3.07*10^4^ |
| MSMEG_4217 | Rv2145c (wag31) | DivIVA family protein; controls growth and polar wall synthesis; protects PbpB from proteolysis | -1.87 | 1.70*10^21^ |
| MSMEG_0026 | Rv0011c (crgA) | facilitates cell separation | 2.04 | 9.40*10^7^ |
| MSMEG_6171 | Rv3660c (SSD) | MinD/Ssd family protein; regulates septum placement and division | -3.2 | 0.02 |
| AG precursor formation | | | | |
| MSMEG_0946 | Rv0501 (galE2) | UDP-glucose 4-epimerase | -1.43 | 1.41*10^16^ |

**Table S4.** NapM interactome identified in pull-down experiment. Bolded hits are the ones which gene transcripts were detected as DEGs in RNA-Seq analysis of *M. smegmatis* ∆*napM* strain.

|  | | **Protein** | | | **Mean (n=4)** | | | |
| --- | --- | --- | --- | --- | --- | --- | --- | --- |
| **Accession** | **Gene annotation** | | **Description** | **Hit count** | **Score** | **Matched  fragments** | **Matched  peptides** | **Sequence  Coverage (%)** |
| **A0R7G5** | **MSMEG_6903** | | **Transcriptional regulator PadR family protein** | **4** | **34800** | **433** | **25** | **67** |
| A0R2Q5 | MSMEG_5197 | | Acyl-acyl-carrier-protein dehydrogenase MbtN | 4 | 6870 | 140 | 16 | 47 |
| A0QWT1 | MSMEG_3053 | | DNA-directed RNA polymerase subunit omega | 2 | 4417 | 21 | 3 | 22 |
| A0QTR2 | MSMEG_1934 | | ATP-binding protein | 2 | 4177 | 10 | 1 | 10 |
| A0QV38 | MSMEG_2436 | | RNA-binding protein KhpA | 3 | 3765 | 45 | 6 | 51 |
| A0R156 | MSMEG_4631 | | DUF937 domain-containing protein | 2 | 3186 | 16 | 1 | 8 |
| A0R1A7 | MSMEG_4684 | | Ribose-5-phosphate isomerase B | 2 | 2926 | 22 | 3 | 28 |
| **A0QQC5** | **MSMEG_0706** | | **Putative 4-hydroxy-4-methyl-2-oxoglutarate aldolase** | **3** | **2843** | **28** | **3** | **20** |
| A0QZ47 | MSMEG_3895 | | Proteasome subunit beta | 2 | 2822 | 26 | 3 | 17 |
| A0QXT5 | MSMEG_3419 | | Ferritin-like domain-containing protein | 2 | 2821 | 39 | 6 | 23 |
| **A0QRD4** | **MSMEG_1076** | | **Acid stress chaperone HdeA** | **2** | **2477** | **7** | **1** | **11** |
| A0QSL9 | MSMEG_1525 | | Large ribosomal subunit protein bL17 | 2 | 2376 | 70 | 6 | 21 |
| A0QTP2 | MSMEG_1914 | | ECF RNA polymerase sigma factor SigH | 2 | 2102 | 21 | 4 | 25 |
| A0QS41 | MSMEG_1341 | | MaoC family protein | 2 | 1986 | 13 | 2 | 21 |
| A0QWH1 | MSMEG_2940 | | Probable transcriptional regulatory protein | 2 | 1767 | 16 | 1 | 8 |
| A0QV18 | MSMEG_2416 | | Cell division initiation protein SepIVA | 2 | 1735 | 23 | 4 | 18 |
| **A0QVT5** | **MSMEG_2688** | | **Antibiotic biosynthesis monooxygenase domain protein** | **2** | **1689** | **6** | **1** | **16** |
| A0R560 | MSMEG_6076 | | 2-C-methyl-D-erythritol 4-phosphate cytidylyltransferase | 2 | 1679 | 11 | 2 | 10 |
| A0R2X3 | MSMEG_5265 | | Cystathionine gamma-synthase | 2 | 1668 | 24 | 3 | 15 |
| A0QTS9 | MSMEG_1951 | | Conserved domain protein | 2 | 1472 | 29 | 4 | 16 |
| A0R062 | MSMEG_4273 | | Integral membrane protein | 4 | 1464 | 50 | 8 | 37 |
| A0QV42 | MSMEG_2440 | | Large ribosomal subunit protein bL19 | 3 | 1425 | 18 | 3 | 38 |
| A0QNF5 | MSMEG_0023 | | Cell wall synthesis protein CwsA | 2 | 1289 | 10 | 1 | 14 |
| A0QV17 | MSMEG_2415 | | Hemerythrin HHE cation binding region | 2 | 1284 | 17 | 4 | 21 |
| A0R5R1 | MSMEG_6280 | | Nucleoid-associated protein | 2 | 1232 | 5 | 1 | 10 |
| A0R678 | MSMEG_6452 | | NADP+ succinate-semialdehyde dehydrogenase | 2 | 1158 | 28 | 5 | 15 |
| A0QX96 | MSMEG_3220 | | Tryptophan synthase beta chain | 2 | 1152 | 18 | 3 | 9 |
| A0QSU3 | MSMEG_1602 | | Inosine-5-monophosphate dehydrogenase | 2 | 1138 | 28 | 5 | 13 |
| **A0QX85** | **MSMEG_3209** | | **Phosphoribosyl isomerase A** | **2** | **1117** | **18** | **4** | **19** |
| A0R566 | MSMEG_6082 | | Carbonic anhydrase | 2 | 974 | 24 | 4 | 25 |
| A0QQL0 | MSMEG_0793 | | Thiazole synthase | 2 | 974 | 21 | 4 | 15 |
| **A0QTK6** | **MSMEG_1878** | | **Ribosome hibernation promoting factor** | **2** | **933** | **23** | **4** | **22** |
| A0QV37 | MSMEG_2435 | | Small ribosomal subunit protein bS16 | 2 | 918 | 15 | 2 | 21 |
| A0QV09 | MSMEG_2407 | | Aldo-keto reductase | 2 | 901 | 20 | 3 | 14 |
| A0QV12 | MSMEG_2410 | | Serine-threonine protein kinase | 2 | 876 | 20 | 4 | 21 |
| A0QXB9 | MSMEG_3246 | | Response regulator | 2 | 856 | 18 | 3 | 23 |
| **A0R343** | **MSMEG_5336** | | **Amidate substrates transporter protein** | **2** | **840** | **7** | **1** | **4** |
| A0QS96 | MSMEG_1398 | | Small ribosomal subunit protein uS12 | 2 | 802 | 6 | 1 | 8 |
| A0R5V7 | MSMEG_6328 | | tRNA adenosine deaminase | 2 | 754 | 7 | 1 | 6 |
| A0QZ46 | MSMEG_3894 | | Proteasome subunit alpha | 2 | 705 | 18 | 3 | 14 |
| A0QYD4 | MSMEG_3619 | | Short chain dehydrogenase | 2 | 683 | 15 | 3 | 14 |
| A0QVU5 | MSMEG_2698 | | Limonene-1 2-epoxide hydrolase domain-containing protein | 2 | 677 | 6 | 1 | 4 |
| A0QQ65 | MSMEG_0643 | | Extracellular solute-binding protein family protein 5 putative | 2 | 656 | 30 | 5 | 13 |
| A0QQP8 | MSMEG_0832 | | Peptide deformylase | 2 | 598 | 11 | 2 | 15 |
| A0QQD0 | MSMEG_0711 | | Chaperone protein DnaJ | 2 | 585 | 13 | 2 | 6 |
| A0R1B6 | MSMEG_4693 | | Uncharacterized protein | 2 | 559 | 5 | 1 | 11 |
| A0R6G6 | MSMEG_6541 | | Anti-sigma factor antagonist | 2 | 558 | 5 | 1 | 10 |
| **A0R729** | **MSMEG_6759** | | **Glycerol kinase** | **2** | **553** | **25** | **6** | **19** |
| A0QWY3 | MSMEG_3106 | | Quinone oxidoreductase | 2 | 546 | 15 | 2 | 11 |
| A0QX32 | MSMEG_3155 | | Band 7 protein | 2 | 542 | 15 | 3 | 9 |
| A0QR91 | MSMEG_1030 | | Monooxygenase | 2 | 523 | 22 | 4 | 9 |
| A0QYU8 | MSMEG_3793 | | Translation initiation factor IF-3 | 2 | 521 | 10 | 1 | 8 |
| A0R203 | MSMEG_4939 | | ATP synthase subunit b-delta | 2 | 497 | 7 | 1 | 2 |
| A0QW43 | MSMEG_2800 | | NADPH-dependent fmn reductase | 2 | 494 | 5 | 1 | 5 |
| A0QZ58 | MSMEG_3906 | | tRNA (adenine(58)-N(1))-methyltransferase TrmI | 2 | 492 | 12 | 3 | 14 |
| A0R1E4 | MSMEG_4722 | | Short-chain dehydrogenase | 2 | 476 | 15 | 4 | 17 |
| A0QPV9 | MSMEG_0536 | | Intracellular protease PfpI family protein | 2 | 474 | 7 | 1 | 12 |
| A0QQQ0 | MSMEG_0834 | | Tuberculin related peptide | 2 | 470 | 9 | 1 | 8 |
| A0R2A4 | MSMEG_5042 | | ATP-dependent RNA helicase DeaD | 2 | 425 | 16 | 4 | 5 |
| **A0QV10** | **MSMEG_2408** | | **Aldo-keto reductase** | **2** | **417** | **12** | **2** | **11** |
| A0QWT7 | MSMEG_3059 | | Esterase | 2 | 410 | 11 | 2 | 7 |
| A0R7F6 | MSMEG_6894 | | Large ribosomal subunit protein bL9 | 2 | 400 | 7 | 2 | 10 |
| A0R4H6 | MSMEG_5837 | | Glutathione peroxidase | 2 | 384 | 7 | 1 | 8 |
| A0R5K8 | MSMEG_6227 | | Transcriptional regulator PadR family protein | 3 | 384 | 8 | 2 | 5 |
| A0QYH7 | MSMEG_3662 | | Mannose-binding lectin | 2 | 380 | 13 | 3 | 14 |
| A0R7J0 | MSMEG_6934 | | Thioredoxin | 2 | 379 | 3 | 1 | 5 |
| A0R2T3 | MSMEG_5225 | | Lipid droplet-associated protein | 2 | 379 | 8 | 2 | 10 |
| A0QTT5 | MSMEG_1957 | | Hydrolase | 2 | 375 | 23 | 5 | 17 |
| A0QQY3 | MSMEG_0918 | | Transcriptional regulator XRE family protein | 2 | 352 | 7 | 2 | 11 |
| A0QWQ4 | MSMEG_3025 | | Alanine--tRNA ligase | 2 | 347 | 17 | 5 | 3 |
| A0R576 | MSMEG_6092 | | Lsr2 protein | 2 | 336 | 12 | 2 | 10 |
| **A0QV23** | **MSMEG_2421** | | **OsmC-like protein** | **2** | **332** | **3** | **1** | **4** |
| A0QV28 | MSMEG_2426 | | Nitrogen regulatory protein P-II | 2 | 321 | 5 | 1 | 10 |
| A0QP11 | MSMEG_0234 | | Metallopeptidase | 2 | 305 | 24 | 7 | 11 |
| A0QVX3 | MSMEG_2727 | | Glutamate binding protein | 2 | 300 | 12 | 3 | 13 |
| A0QVR8 | MSMEG_2669 | | Hydrolase | 2 | 298 | 7 | 1 | 8 |
| A0QX03 | MSMEG_3126 | | SUF system FeS assembly protein NifU family protein | 2 | 292 | 4 | 1 | 4 |
| P71534 | MSMEG_3150 | | 3-oxoacyl-acyl-carrier-protein reductase MabA | 2 | 289 | 6 | 1 | 8 |
| A0QQF9 | MSMEG_0741 | | Luciferase-like domain-containing protein | 2 | 266 | 7 | 2 | 5 |
| A0R4J1 | MSMEG_5852 | | Phosphoribosylamine--glycine ligase | 2 | 260 | 11 | 4 | 10 |
| A0QNX2 | MSMEG_0194 | | Serine esterase cutinase family protein | 2 | 256 | 4 | 1 | 7 |
| A0R2D2 | MSMEG_5070 | | Trypsin | 2 | 238 | 15 | 4 | 8 |
| A0QR08 | MSMEG_0943 | | Pyrroline-5-carboxylate reductase | 2 | 226 | 13 | 3 | 15 |
| A0R3L1 | MSMEG_5512 | | Magnesium chelatase | 2 | 217 | 12 | 3 | 8 |
| A0R2D5 | MSMEG_5073 | | Putative O-methyltransferase | 2 | 213 | 5 | 1 | 6 |
| A0QQF0 | MSMEG_0732 | | Chaperone protein ClpB | 2 | 208 | 20 | 6 | 6 |

**Table S5.** Oligonucleotides used in this study.

| **Name** | **Sequence** | **Application** |
| --- | --- | --- |
| Ms_attB_L5_down | AGGCACATGCTGCCACTG | Construction of *M. smegmatis mc^2^* mutant strains (see Text S1). |
| Ms_attB_L5_up | AGCGGATGCGCTACCAAG |  |
| NapM1_Fw | TAACTGTGATAAACTACCGCATTAAGTGGCAACGCGGCAG |  |
| NapM1_Rv | CCGACATAAGCTTTGGATTGTCCGTACGCCCC |  |
| NapM2_Fw | AAATAAGCTAGCACGCACCCCGGGTAC |  |
| NapM2_Rv | ATTACAGGGGAATTCTTAATTAAGCTCAGCCCTCGATGAAGGTTTCCA |  |
| mNG_Fw | ACGGACAATCCAAAGCTTATGTCGGCTGGCT |  |
| mNG_Rv | ACCCGGGGTGCGTGCTAGCTTATTTGTACAATTCATCCATGCC |  |
| NapM1_ver_Fw | GTGGCAACGCGGCAGACG |  |
| NapM2_ver_Rv | TCAGCCCTCGATGAAGGTTTC |  |
| napM1del_RV | ACCCGGGGTGCGTCCCCTATCCAGCTCTGTTCGG |  |
| napM2del_Fw | GCTGGATAGGGGACGCACCCCGGGTACA |  |
| HindIII_Flagx3_FW | AGCTTGACTACAAGGACGATGACGACAAGGACTACAAGGACGATGACGACAAGGACTACAAGGACGATGACGACAAGTGAG |  |
| NheI_Flagx3_RV | CTAGCTCACTTGTCGTCATCGTCCTTGTAGTCCTTGTCGTCATCGTCCTTGTAGTCCTTGTCGTCATCGTCCTTGTAGTCA |  |
| Flag_ver_Rv | TTACTT GTCGTC ATCGTC CT |  |
| pMV_pami__napM_FW | GAGGTAGTTTTCGGATCCAGTACTTCTAGAGTGATTCCGGTGCTGGAG |  |
| pMV_pami__napM_RV | CCGACATCATATGTGGATTGTCCGTACGCCCCTG |  |
| pMV_pami___mNG_FW | ACGGACAATCCACATATGATGTCGGCTGGCTC |  |
| pMV_pami__mNG_RV | CCGAACGACCGAGCGCAACGCGTGCGGCCGCTTATTTGTACAATTC |  |
| BTH_xbaI_napM_Fw | TCTAGAAGTGATTCCGGTGCTGGAGC | Construction of *E. coli* BTH101 strains |
| BTH_kpnI_napM_Rv | GGTACCTTTGGATTGTCCGTACGC |  |
| BTH_napM_domenaN_kpnI_Rv | ATGGGT ACCTTA CCGAAG CCGTCG TCGGAG TAGTT |  |
| BTH_napM_domenaC_XbaI_Fw | ATGTCT AGAAAA CTACTC CGACGA CGGCTT CG |  |
| pACYC_napM_Fw | GTTTAACTTTAATAAGGAGATATACATGATTCCGGTGCTGGAGC | Construction of *E. coli* BL21 (DE3) strain |
| pACYC_mNG_NheI_Rv | TCGACTTAAGCATTATGCGGCCGCAGCTAGCTTATTTGTACAATTCATCCATGCC |  |
| T7 promotor | TAATAC GACTCA CTATAG GG |  |
| T7 terminator | GCTAGTTATTGCTCAGCGG |  |

**Table S6. Plasmids used in this study.**

| **Name** | **Plasmid feature** | **Reference** |
| --- | --- | --- |
| p2NIL Ø | kanamycin resistance, *oriE*, suicide plasmid for allelic replacement | (Parish & Roberts Editors, 2015) |
| pGOAL17 Ø | ampicillin resistance, *oriE*, selective PacI selective cassete with *lacZ*, *sacB* and *kanR* genes | (Parish & Roberts Editors, 2015) |
| pMV_pAMI_ Ø | kanamycin resistance, *oriE*, inducible promotor *p_AMI_, attB* integrative plasmid for mycobacterial transformation | Lab collection |
| pACYCDuet™-1 | CmR, *cat* promoter, p15A *ori*, *lac* promotor, repressor, operator, His-tag, S-tag | Lab collection |
| pKNT25 Ø | kanamycin resistance, p15A *ori*, *lacZ*, CAP binding site, T25 fragment of *cya* | Euromedex |
| pKT25 Ø | kanamycin resistance, p15A *ori*, *lacZ*, CAP binding site, T25 fragment of *cya* | Euromedex |
| pUT18 Ø | ampicilin resistance, p15A *ori*, *lacZ*, CAP binding site, T18 fragment of *cya* | Euromedex |
| pUT18C Ø | ampicilin resistance, p15A *ori*, *lacZ*, CAP binding site, T18 fragment of *cya* | Euromedex |
| p2NIL ∆NapM | plasmid constructed on p2NIL Ø backbone with inserted flanking regions of *napM* | this study |
| p2NIL ∆NapM GOAL | plasmid constructed on p2NIL delta NapM backbone with inserted *goal* cassette from pGOAL17 Ø. | this study |
| p2NIL NapM-FLAGx3 | plasmid constructed on p2NIL Ø backbone with inserted *napM-flagx3* | this study |
| p2NIL NapM-FLAGx3 GOAL | plasmid constructed on p2NIL NapM Flagx3 backbone with inserted *goal* cassette from pGOAL17 Ø. | this study |
| p2NIL NapM mNG | plasmid constructed on p2NIL Ø backbone with inserted *napM-mNeoGreen* | this study |
| p2NIL NapM-mNeonGreen GOAL | plasmid constructed on p2NIL *napM-mneongreen* backbone with inserted *goal* cassette from pGOAL17 Ø. | this study |
| pACYCDuet™-1 NapM-mNeonGreen | plasmid constructed on pACYCDuet™-1 backbone with inserted *napM-mneongreen* | this study |
| pKNT25 NapM | plasmid constructed on pKNT25 Ø backbone with inserted *napM* | this study |
| pKT25 NapM | plasmid constructed on pKT25 Ø backbone with inserted *napM* | this study |
| pUT18 NapM | plasmid constructed on pUT18C Ø backbone with inserted *napM* | this study |
| pUT18C NapM | plasmid constructed on pUT18 Ø backbone with inserted *napM* | this study |
| pUT18C DivIVA | plasmid constructed on pUT18C Ø backbone with inserted *divIVa* | (Ginda et al., 2013) |
| pUT18 DivIVA | plasmid constructed on pUT18 Ø backbone with inserted *divIVa* | (Ginda et al., 2013) |
| pMV_pAMI_ NapM-mNeonGreen | plasmid constructed on pMV_pAMI_ Ø backbone with inserted *napM-mneongreen* | this study |
| pMV_pAMI_ napM-FLAGx3 | plasmid constructed on pMV_pAMI_ Ø backbone with inserted *napM* with *flagx3* tag | this study |
| pMV_pMS_ DivIVA-mCherry | Plasmid constructed on pMV_306_ Ø backbone with inserted promotor of DivIVA and DivIVA-mCherry | (Ginda et al., 2013) |

**Table S7.** Strains used in this study.

| **Name** | **Relevant genotype** | **Source** |
| --- | --- | --- |
| WT | *M. smegmatis mc^2^ 155* | Lab collection |
| Control↑ | *M. smegmatis mc^2^ 155 attBL5::*pMV306_pAMI_ *Ø* | Lab collection |
| DnaN-mCherry | *M. smegmatis mc^2^ 155 dnaN-mcherry* | Lab collection |
| NapM-mNeonGreen | *M. smegmatis mc^2^ 155 napM-mneongreen* | This study |
| ∆napM | *M. smegmatis mc2 155 ∆napM* | This study |
| NapM-mNeonGreen↑ | *M. smegmatis mc^2^ 155 napM-mneongreen, attBL5::*pMV306_pAMI_ *napM-mneongreen* | This study |
| NapM↑ | *M. smegmatis mc^2^ 155 attBL5::*pMV306_pAMI_ *napM* | This study |
| ∆napM/DnaN-mCherry | *M. smegmatis mc^2^ 155 ∆napM, dnaN-mcherry* | This study |
| NapM-FLAGx3 | *M. smegmatis mc^2^ 155 napM-flagx3* | This study |
| NapM-FLAGx3↑ | *M. smegmatis mc^2^ 155 attBL5::*pMV306_pAMI_ *napM-flagx3* | This study |
| NapM-mNeonGreen/DivIVA-mCherry | *M. smegmatis mc^2^ 155 napM-mneongreen, attBL5::*pMV306_pMS_ *divIVa-mcherry* | This study |
| DivIVA-mCherry | *M. smegmatis mc^2^ 155, attBL5::*pMV306_pMS_ *divIVa-mcherry* | Lab collection |
| NapM-mNeonGreen_Ec_ | *E. coli BL21 (DE3)* pACYC *napM-mneongreen* | This study |
| pACYC Ø | *E. coli BL21 (DE3)* pACYC *Ø* | This study |
