# Supplementary material for: The mycobacterial nucleoid-associated protein NapM exhibits stress-induced septal localization and modulates cell envelope gene expression
Source: Microbiol Spectr. 2026 Jun 9;14(7):e03938-25. doi: 10.1128/spectrum.03938-25 (PMC13340245; doi:10.1128/spectrum.03938-25)
Supplement: Supplemental material — Experimental procedures. [file spectrum.03938-25-s0005.docx]

# **Text S1. Supplementary Experimental Procedures**

## **Plasmid Propagation and Bacteria Cultivation**

Plasmids utilized for *Mycobacterium smegmatis* *mc^2^ 155* transformation were propagated in *Escherichia coli* DH5α. *E. coli* was cultured in Luria-Bertani (LB) broth at 37°C with agitation at 180 rpm and on LB agar plates (Difco) at 37°C (1). Media were supplemented with antibiotics (100 µg/ml ampicillin, 50 µg/ml kanamycin) or other additives if needed, including 0.004% X-Gal [5-bromo-4-chloro-3-indolyl-α-D-galactopyranoside]. *M. smegmatis* liquid cultures were grown in Middlebrook 7H9 supplemented with 0.05% Tween 80 and albumin-dextrose-catalase (ADC; BD) medium, or in Difco Nutrient Broth (NB; BD) medium at 37°C with agitation at 180 rpm. Solid medium cultivation involved the addition of 2% agar to NB or Middlebrook 7H10 medium with oleic acid-albumin-dextrose-catalse (OADC; BD). Cultures were incubated at 37°C until visible colony formation occurred within 2-5 days. Antibiotics (100 µg/ml ampicillin, 50 µg/ml kanamycin, 2.5–7.5 µg/ml rifampicin, 0.4 µg/ml ethambutol, 5 µg/ml novobiocin, 6 µM benzyldodecyldimethylammonium chloride (DDBAC, the C12 chain variant of benzalkonium chloride; referred to throughout the manuscript as BAC), 0.75 µg nisin, triclosan 0.1 µg/ml, vancomycin 2 µg/ml, carbenicillin 250 µg/ml) and other additives (0.004% X-Gal, 2% sucrose,1 mM Isopropyl β-D-1-thiogalactopyranoside (IPTG), 1% acetamide) were added if required.

## **Construction *E. coli* BL21 (DE3) expressing NapM-mNeonGreen**

To visualize NapM in *E. coli* cells, the PCR-amplified product, generated using primers pACYC_napM_Fw and pACYC_mNG_NheI_Rv (**Table S3**), was cloned into the NcoI and HindIII sites of the pACYCDuet™-1 vector (Sigma). Transformants were selected on LB agar supplemented with chloramphenicol. The resulting plasmid was verified by PCR and sequencing, and transformed into *E. coli* BL21(DE3) cells. Positive clones were used in fluorescence microscopy experiments. An overnight culture of the transformants was grown in LB liquid medium supplemented with chloramphenicol and a small portion of the culture was used to inoculate fresh medium. Once the culture reached an OD_600_ of 0.4–0.6, isopropyl β-D-1-thiogalactopyranoside (IPTG) was added to a final concentration of 1 mM, and the culture was incubated for 1 hour. Subsequently, 1 ml of the culture was stained with the chromosomal dye DAPI (Molecular Probes) at a final concentration of 2 µg/ml for 20 minutes. Cells were centrifuged at 5000 × g for 5 minutes, washed with phosphate-buffered saline (PBS), and resuspended in PBS. The suspension was smeared onto microscopic slides. As a negative control, *E. coli* BL21(DE3) cells transformed with an empty pACYCDuet™-1 vector were used.

## **Construction of *M. smegmatis mc^2^* 155 mutant strains**

The allelic replacement of *napM* gene (*MSMEG_6903*) with fusion genes *napM-mneongreen or napM-flag* was performed following the protocol outlined by Parish and Roberts (2). Briefly, the chromosome of *M. smegmatis* *mc^2^ 155* was used as template to amplify downstream and upstream regions of *napM* gene using two primer sets napM1_FW x napM1_RV and napM2_FW x napM2_RV. In the case of *mneongreen* fusion gene, it was amplified by PCR utilizing primer sets - mNG_FW x mNG_RV. Amplified products were cloned to p2NIL Ø plasmid and transformed colonies were spread on LB medium supplemented with kanamycin. In the case of *napM-flagx3* fusion, *flagx3* complementary oligonucleotides (Genomed) - HindIII_Flagx3_FW x NheI_Flagx3_RV with HindIII and NheI overhangs were annealed and cloned to p2NIL *napM-mneongreen* digested with HindIII and NheI restriction enzymes.

Analogous cloning strategy was used for construction of *M. smegmatis mc^2^ 155* strain with deletion of *napM* gene (*MSMEG_6903*). Flanking regions of *MSMEG_6903* were amplified using primers sets - napM1_FW x napM1del_RV and napM2del_FW x napM2_RV. Obtained products were cloned to p2NIL Ø plasmid and transformants were plated on LB supplemented with kanamycin.

In the final step, *goal* cassette was cloned into PacI site of every p2NIL derivative. Electrocompetent *M. smegmatis* cells were electroporated with 0,5 µg – 8000 µg of NaOH/EDTA-treated plasmid DNA and unmarked mutants were selected according to the procedure described previously by Parish and Roberts (2). In short, after transformation, cells were plated on NB supplemented with kanamycin and X-gal. Blue single crossing-over (SCO) mutants were further spread for biomass (on NB plates supplemented with 2% sucrose and X-gal) which was then induced to second crossing-over. Chosen double crossing-over (DCO) mutants were verified by PCR and sequencing. If applicable, Western blotting was used to prove the production of fusion proteins.

In the case of pMV_pAMI_ Ø integrative plasmids carrying fusions of *napM* gene, the following primers were used - pMV_pAMI__napM_FW x pMV_pAMI__napM_RV and pMV_pAMI__mNG_FW x pMV_pAMI__mNG_RV for *napM-mneongreen*. Amplified products were cloned using SLIC (3) to pMV_pAMI_ Ø plasmid. Obtained derivatives of pMV_pAMI_ Ø plasmid were used to transform electrocompetent *M. smegmatis* cells. Transformants were spread on NB medium supplemented with kanamycin and colonies were verified by PCR.

To compare growth of the constructed *M. smegmatis* *mc^2^ 155* strains, cells were grown at 37˚C in a final volume of 300 μl 7H9 (supplemented with ADC and 0.05% Tween 80, and 1% acetamide if applicable). Optical density measurements were taken at 20 min intervals for 30 – 60 h using a Bioscreen C instrument. To determine the differences in growth rates of the analyzed strains, OD_600_ values within the linear range of the growth curves were log10-transformed, and the slopes of the resulting curves were used to compare the growth rates.

## **Western blotting**

Western blotting analysis was performed to assess the levels of fusion proteins in NapM-FLAG, NapM-mNeonGreen, and NapM-mNeonGreen↑ strains under various conditions. Exponential phase cells (OD_600_ = 0,8) were centrifuged, and pellet was sonicated in PBS to prepare cell lysates. For SDS-PAGE 20 - 100 µg of the total protein of each lysate were used. Western blotting was performed by transferring proteins to a nitrocellulose membrane. The membrane was blocked with 5% non-fat dry milk in TBST (TBS + 0.1% Tween-20) overnight at 4°C to prevent non-specific binding. The primary antibodies used were an anti-FLAG antibody (Sigma, dilution 1:1000) for NapM-FLAG and an anti-mNeonGreen antibody (ChromoTek, dilution 1:1000) for NapM-mNeonGreen and NapM-mNeonGreen↑ strains. The membranes were incubated with the primary antibody for 1 h at room temperature. After washing with TBST, membranes were incubated with goat anti-mouse IgG secondary antibody conjugated with HRP (Invitrogen, dilution 1:5000) for 1 h at room temperature. Protein bands were visualized using Pierce™ SuperSignal™ West Pico PLUS Chemiluminescent Substrate (Thermo Scientific) and detected with a chemiluminescence imaging system (ChemiDoc MP, Bio-Rad). Loading control was performed using Ponceau S Staining solution accordingly to the procedure provided by the manufacturer (ThermoFisher Scientific).

## **Two-dimensional thin-layer chromatography (2D-TLC)**

2D TLC was performed with 300 µg of total lipids for each strain on HPTLC plates, which were developed in two directions: the first in the solvent system containing chloroform, methanol and water (65:25:4, v/v/v) and the second containing chloroform, acetic acid, methanol and water (80:15:12:4, v/v/v/v). The HPTLC plates were revealed with 0.5% (w/v) vanillin in ethanol with 3% H_2_SO_4_, followed by heating the plate at 120 °C. The vanillin reagent enabled the visualization of lipids (4).

## **TLC of mycolic acid methyl esters (MAMES)**

Mycolic acids were obtained from dry cell mass by an alkaline method using 15% tetrabutylammonium hydroxide and then analyzed by TLC (4). The mycolic acid methyl esters were dissolved in chloroform in the concentration of 50 mg/ml and applied on silica gel 60 TLC plates (Merck). Lipids were analyzed by TLC in a solvent system: hexane-diethyl ether (85:15, v/v) developed three times and were visualized by 10% molybdophosphoric acid in an ethanol solution, followed by heating at 120°C (4).

## **Pull-down assay**

To perform pull-down experiment, liquid cultures of NapM-FLAG and WT strains (OD_600_ = 0.8) were used. Cells were harvested by centrifugation at 5,000 RPM for 20 minutes at 4°C, washed twice with ice-cold PBS, and resuspended in 10 mL of freshly prepared immunoprecipitation (IP) buffer (50 mM Tris-HCl pH 8.0, 250 mM NaCl, 0.8% Triton X-100) supplemented with protease inhibitor tablets (PierceTM Protease Inhibitor, ThermoScientific, A32965). Cells were then lysed by sonication (25 minutes total, 5 seconds on / 5 seconds off cycles, 50% amplitude) on ice. Lysates were centrifuged at 10,000 RPM for 20 minutes at 4°C, and collected supernatant was centrifuged once more to ensure complete removal of cell debris. The quantification of total protein within lysates were performed using Bradford method with ROTI®Quant (K015.1, Roth). For pull-down experiment 10 mg of total protein was used in final volume of 14 ml IP Buffer. 100 µl of magnetic anti-FLAG beads (PierceTM ANTI-DYKDDDDK Magnetic Agarose, ThermoScientific, A36797) were washed three times in IP buffer to remove storage buffer, added to the lysate and incubated overnight at 4°C on a rotating platform. Then, beads were collected using a magnetic stand and washed four times with 1 mL of cold IP buffer.

To prepare samples for mass spectrometry analysis, beads were washed four times in 25 mM Tris pH 7.5. Bead-bound proteins were then denatured at 65°C for 10 minutes in 25 mM Tris pH 7.5, 0.1% RapiGest SF (RGSF), 3 mM DTT. Subsequently, 100 ng of trypsin was added to the sample for an overnight on-bead digestion in 37°C. Next day the solution was separated from the beads, acidified and RGSF was removed by centrifugation. The supernatant was then desalted using a STAGE tip (5). Obtained peptide pellet was resuspended in 0.1% formic acid (FA), 3% acetonitrile (ACN) solution.

## **Liquid chromatography–Mass spectrometry (LC-MS) sample preparation and analysis**

LC-MS was performed on an M-Class Acquity UPLC connected to a Synapt XS HDMS equipped with a nanoESI source. Mobile phase A consisted of H_2_O + 0.1% FA, while mobile phase B contained ACN + 0.1% FA. A 5–35%. 60 min linear gradient at a 300 nL/min flow rate was applied for sample separation on a C18 75 μm x 250 mm analytical column kept at 45°C. A 5-minute sample trapping step was performed prior to analytical column separation. MS data were collected in ion mobility DIA (HDMSE) at a scan rate of 0.6 seconds. Source conditions were fine-tuned as follows: Capillary (Picotip, Waters): 2.5 kV; Sampling Cone: 35 V; Source Offset: 15 V; Source temperature: 80°C; cone gas flow: 40 L/h, purge gas flow: 100 L/h, NanoFlow gas pressure: 0.1 Bar. For MS2, a collision energy ramp of 27–47 V was set on the transfer cell. A (Glu1)-Fibrinopeptide B solution was acquired in-parallel as mass reference, and correction was applied post-acquisition.

Raw data processing was performed using Progenesis QiP v4.2.7 (Non-linear Dynamics). QiP’s autovalues were unchanged for the deconvolution, and the auto-optimization of low and high energy ion thresholds was chosen. Deconvoluted data were searched via Ion Accounting against a *Mycobacterium smegmatis* protein sequence databank (UP000000757) to which porcine trypsin, human keratins, rat Ig gamma-2A C and Ig lambda-2 C sequences were appended (UniProt entries). Rat Ig sequences were identified in a SwissProt 1.0 databank pre-search, and are most likely resin related. The search parameters were as follows; peptide mass tolerance: 15 ppm; fragment mass tolerance: 25 ppm; min. fragments/peptide: 1; min. fragments/protein: 3; min. peptides/protein: 1; max. protein mass: 1 MDa; digest reagent: trypsin; max. missed cleavages: 4; variable modification: oxidation of methionine; FDR: 1%. Protein grouping was enabled.

The Ion Accounting final protein list output was obtained and analyzed for each sample. The mean score and hit count across biological replicates (n = 4 for NapM-FLAG, n = 3 for WT) for each identified protein were calculated. The protein list was filtered to hits identified in at least two NapM replicates and not present in any WT replicate. These proteins were considered as potential NapM partners.

The mass spectrometry proteomics data were deposited to the ProteomeXchange Consortium via the PRIDE (6) partner repository with the dataset identifier PXD062473.

# **Bibliography**

1. Sambrook J, W. Russel D. 2001. Molecular Cloning: A Labortaroy ManualSociety.

2. Parish T, Roberts Editors DM. 2015. Mycobacteria Protocols. Springer New York, New York, NY. http://www.springer.com/series/7651.

3. Li MZ, Elledge SJ. 2007. Harnessing homologous recombination in vitro to generate recombinant DNA via SLIC. Nat Methods 4:251–256.

4. Embley M. 1994. Structural Lipids of eubacteria. Chemical methods in prokaryotic systematics 121–162.

5. Rappsilber J, Ishihama Y, Mann M. 2003. Stop and Go Extraction Tips for Matrix-Assisted Laser Desorption/Ionization, Nanoelectrospray, and LC/MS Sample Pretreatment in Proteomics. Anal Chem 75:663–670.

6. Perez-Riverol Y, Bai J, Bandla C, García-Seisdedos D, Hewapathirana S, Kamatchinathan S, Kundu DJ, Prakash A, Frericks-Zipper A, Eisenacher M, Walzer M, Wang S, Brazma A, Vizcaíno JA. 2022. The PRIDE database resources in 2022: a hub for mass spectrometry-based proteomics evidences. Nucleic Acids Res 50:D543–D552.

**Legend**

**Figure S1.** Phylogenetic relationships and sequence diversity of NapM homologs.

**Figure S2.** Homodimerization of NapM.

**Figure S3.** Phenotypic analysis of the wild-type (WT) and *napM* mutant strains.

**Figure S4.** Analysis of replication dynamics in Δ*napM* and wild-type (WT) strains.

**Figure S5.** Thin layer chromatography (TLC) profiles of methyl esters of mycolic acids and polar lipids of the wild-type and ∆*napM* strains.

**Figure S6.** Transcriptomic changes in the inositol and phosphatidylinositol biosynthesis pathways in *M. smegmatis* *ΔnapM* strain.

**Figure S7.** Comparative proteomic analysis of NapM-FLAG and WT strains of *M. smegmatis*.

**Figure S8.** Analysis of growth rates in *M. smegmatis* cells overproducing NapM under various stress conditions.

**Figure S9.** Western blot analysis showing upregulation of NapM-FLAG protein upon exposure to stress factors.

**Figure S10.** Colocalization of NapM-mNeonGreen with DAPI-stained nucleoid in *E. coli* cells.

**Figure S11.** Western blot analysis of NapM-mNeonGreen and NapM-mNeonGreen↑ strains.

**Figure S12.** Western blot analysis of NapM-mNeonGreen fusion protein level in NapM-mNeonGreen↑ strain in optimal conditions with different acetamide (inducer) concentrations.

**Figure S13.** Time-lapse imaging of NapM-mNeonGreen↑ cells upon 6 h triclosan exposure.

**Figure S14.** Analysis of NapM-mNeonGreen localization in *M. smegmatis* cells upon ethambutol (EMB) exposure.

**Figure S15.** AlphaFold 3 prediction of tertiary structure of NapM-DivIVA oligomer.

**Table S1.** Differentially expressed genes in *∆napM* strain

**Table S2.** Enriched pathways in ∆*napM* strain

**Table S3.** Comparative analysis of gene expression changes for polar lipid metabolism, mycothiol, and peptidoglycan biosynthesis in *M. smegmatis ∆napM* strain.

**Table S4.** NapM binding partner identified in pull-down experiment.

**Table S5.** Oligonucleotides used in this study.

**Table S6.** Plasmids used in this study.

**Table S7.** Strains used in this study.

**Movie 1.** Time-lapse imaging of NapM–mNeonGreen cells using ONIX microfluidics system during ethambutol (EMB) exposure without antibiotic washout. Arrows indicate septal localization of NapM–mNeonGreen. Images were acquired automatically every 10 min. Scale bar: 5 μm. Time-lapse images were processed in ImageJ using LUT: Smart.

**Movie 2.** Time-lapse imaging of NapM-mNeonGreen↑ cells using ONIX microfluidics system upon 6 h ethambutol (EMB) exposure, followed by antibiotic washing. Yellow arrows indicate septum-like localization of NapM-mNeonGreen. Images were acquired automatically every 10 min. Scale bar:1 μm.

**Movie 3.** Time-lapse imaging of NapM-mNeonGreen cells producing additional copy of DivIVA fused with mCherry using ONIX microfluidics system upon 8 hours ethambutol (EMB) exposure, followed by antibiotic washing. Arrows indicate septal localization of NapM-mNeonGreen (green arrows) or DivIVA-mCherry (red arrows). Images were acquired automatically every 10 min. Scale bar: 1 μm.
